# Discovery, expression, cellular localization, and molecular properties of a novel, alternative spliced HP1γ isoform, lacking the chromoshadow domain

**DOI:** 10.1371/journal.pone.0217452

**Published:** 2020-02-06

**Authors:** Angela Mathison, Thiago Milech De Assuncao, Nikita R. Dsouza, Monique Williams, Michael T. Zimmermann, Raul Urrutia, Gwen Lomberk

**Affiliations:** 1 Genomics and Precision Medicine Center (GSPMC), Medical College of Wisconsin, Milwaukee, Wisconsin, United States of America; 2 Division of Research, Department of Surgery, Medical College of Wisconsin, WI Center, Medical College of Wisconsin, Milwaukee, Wisconsin, United States of America; 3 Bioinformatics Research and Development Laboratory, and Precision Medicine Simulation Unit, Genomics and Precision Medicine Center (GSPMC), Medical College of Wisconsin, Milwaukee, Wisconsin, United States of America; 4 Department of Surgery, Duke University Medical Center, Durham, North Carolina, United States of America; 5 Clinical and Translational Sciences Institute, Medical College of Wisconsin, Milwaukee, Wisconsin, United States of America; 6 Department of Biochemistry, Medical College of Wisconsin, Milwaukee, Wisconsin, United States of America; 7 Department of Pharmacology and Toxicology, Medical College of Wisconsin, Milwaukee, Wisconsin, United States of America; Universität Stuttgart, GERMANY

## Abstract

By reading the H3K9Me3 mark through their N-terminal chromodomain (CD), HP1 proteins play a significant role in cancer-associated processes, including cell proliferation, differentiation, chromosomal stability, and DNA repair. Here, we used a combination of bioinformatics-based methodologies, as well as experimentally-derived datasets, that reveal the existence of a novel short HP1γ (CBX3) isoform, named here sHP1γ, generated by alternative splicing of the *CBX3* locus. The sHP1γ mRNA encodes a protein composed of 101 residues and lacks the C-terminal chromoshadow domain (CSD) that is required for dimerization and heterodimerization in the previously described 183 a. a HP1γ protein. Fold recognition, order-to-disorder calculations, threading, homology-based molecular modeling, docking, and molecular dynamic simulations show that the sHP1γ is comprised of a CD flanked by intrinsically disordered regions (IDRs) with an IDR-CD-IDR domain organization and likely retains the ability to bind to the H3K9Me3. Both qPCR analyses and mRNA-seq data derived from large-scale studies confirmed that sHP1γ mRNA is expressed in the majority of human tissues at approximately constant ratios with the chromoshadow domain containing isoform. However, sHP1γ mRNA levels appear to be dysregulated in different cancer types. Thus, our data supports the notion that, due to the existence of functionally different isoforms, the regulation of HP1γ-mediated functions is more complex than previously anticipated.

## Introduction

HP1 is a cancer-associated chromatin protein, which was first identified as a major component of heterochromatin[1, 2]. HP1 is highly conserved from *S*. *pombe* to mammals, in which three closely related paralogs exist: HP1α, HP1β, and HP1γ[3]. Three HP1 family members in mammals are similar in amino acid sequences and structural organization, but functionally distinct. The structure of HP1 proteins includes an N-terminal chromodomain (CD), followed by a linker region, and then a C-terminal chromoshadow domain (CSD)[4]. HP1 is an elongated molecule in which intrinsically disordered regions (IDRs) allow this protein to have dynamic flexibility, intermolecular recognition properties, and the ability to integrate signals from various intracellular pathways. Extensive structural and biochemical studies have demonstrated that this modular structure is of paramount importance for HP1 proteins to perform their molecular and cellular functions[4–8]. Indeed, HP1 proteins use their CD to bind either the di-methylated or tri-methylated forms of H3K9 within genomic regions that are marked in this manner to be transcriptionally silent[5, 6]. The linker region, which lies in between the CD and CSD domains, contains a nuclear localization signal (NLS) and a DNA binding motif[4, 7, 8]. Finally, the CSD is used by HP1 proteins to homo- or hetero-dimerize among themselves, which is the most common manner to regulate their functional specificity. Notably, CSD dimerization leads to the formation of a docking motif, which is used by HP1 proteins to interact with large variety of chromatin regulators, typically through a PXVXL consensus motif[4]. These additional interactions allow the execution of complex-specific cancer-associated functions, including DNA recombination and repair, cell proliferation, differentiation, and migration, as well as chromosomal stability[4, 9]. Interestingly, studies on HP1 have stimulated the field of epigenomics in a manner that has resulted in the discovery of a larger family of proteins, which, by having similar domains to HP1, were found to display related functions in chromatin remodeling and epigenomics. By analogy to HP1, these CD-containing proteins are collectively known as Chromobox proteins, or CBXs, and are primarily involved in gene silencing[10]. Thus, the significant number of biochemical and biophysical studies that have been focused on defining the structure-function relationship of the different domains within HP1 have advanced our understanding of one of the most important families of proteins involved in epigenomic regulation.

Our laboratory studies the role of HP1-related pathways in pancreatic ductal adenocarcinoma (PDAC), since several members of the pathway are deregulated in this type of aggressive tumor and others[9, 11]. Surprisingly, during the course of RNA-Seq experiments[12], we discovered that the *CBX3* locus, which encodes for HP1γ, undergoes alternative splicing, leading to the generation of the conventional CD-linker-CSD containing HP1γ protein, as well as a novel shorter isoform (sHP1γ). Using qPCR and an anti-sHP1γ antibody, we confirm that, indeed, this spliced isoform is expressed in a variety of tissues and retains the typical nuclear localization expected from its functions. However, while this shorter isoform preserves a CD, it lacks the CSD contained within the conventional longer isoform. Thus, we infer that alternative splicing of the *CBX3* locus gives rise to different proteins, which have distinct structural and molecular properties to likely function in both overlapping and divergent manners.

## Results

### Identification of sHP1γ reveals an expanded repertoire of HP1γ isoforms in human tissues

**Fig 1:**
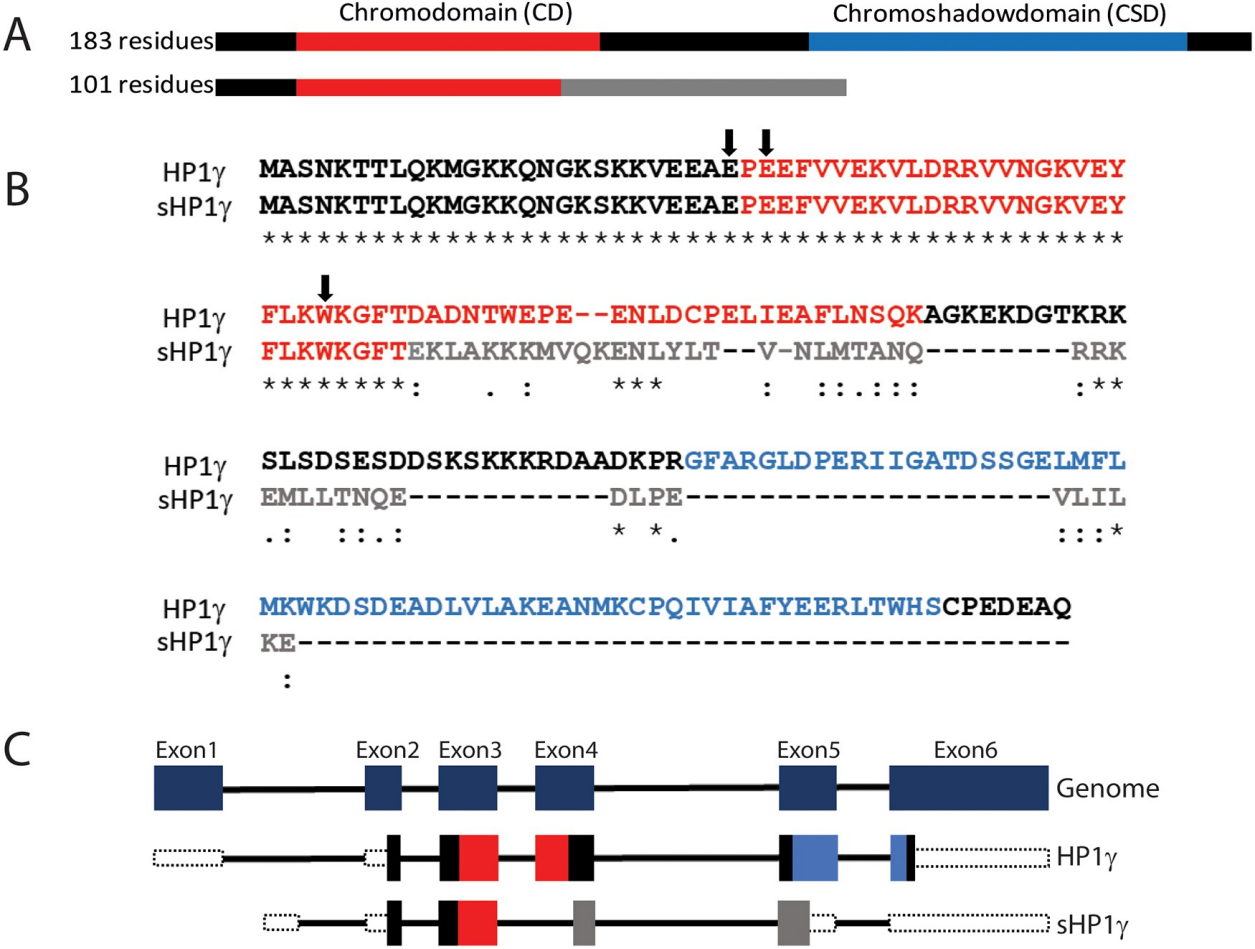
Identification of sHP1γ and comparison with canonical HP1γ. (A) Physical map of a new HP1γ isoform generated by alternative splicing. The conventional HP1γ is 183 residues and contains a chromodomain (CD) and a chromoshadow domain (CSD). A shorter isoform lacks the CSD and is 101 residues. (B) Alignment of sHP1γ protein sequence with HP1γ. The alignment was made using Basic Local Alignment Search Tool (BLAST). Sequences corresponding to the CD and CSD are indicated in red and blue, respectively, while the sequence generated by alternative splicing is depicted in gray. Arrows represent residues involved in histone binding. (C) The HP1γ genomic structure (top) with dark blue boxes representing constitutive exons. The lower panel illustrates mRNA splicing for the canonical and sHP1γ isoforms. CD is represented in red boxes, CSD is represented in blue boxes and sHP1γ sequence generated by alternative splicing is represented in gray boxes.

Our laboratory has made significant contributions to the study of HP1 proteins in transcriptional regulation, epigenetics and cancer. In fact, the current study was derived from initial experiments looking at the expression pattern of HP1 proteins in pancreatic cancer cells. Careful examination of the reads obtained by RNA-Seq[12] suggested that the *CBX3/HP1γ* locus undergoes alternative splicing to give rise to a new protein-coding isoform. The *CBX3* locus is located within human chromosome 7 and has 6 exons, and annotation of these gene transcripts is also found on Ensembl/Havana (ENST00000409747 and OTTHUMT00000327972.1). *CBX3* is currently known to produce a protein-coding transcript 2128bp in length corresponding to a protein of 183 residues. The protein structure follows the typical IDR-CD-IDR-CDS-IDR structure known for all HP1s[4]. Notably, however, in this study, we report the existence of an additional 1717bp transcript which encodes a smaller protein of 101 amino acids that lacks the C-terminal CSD (Fig 1A and 1B), hereafter named short HP1γ (sHP1γ). sHP1γ is generated by a splicing event that skips part of the fourth exon, shifts the reading frame and thus initiates a new stop codon in exon 5 (Fig 1C). Thus, the CSD found at the C-terminus of the conventional HP1γ is absent in the short isoform described here, which makes this protein unique among all members of this family (Fig 1A and 1B). Additional analysis of the primary structure of this protein, using the ProtParam algorithm[13] yields an expected Mw of 11.85796 KDa and a theoretical pI of 9.63, the latter being similar to most nuclear proteins, in particular histones and those which associate to them. Additional key characteristics, calculated from the sHP1γ primary structure, are its extinction coefficient, which informs our ability to measure the concentrations of this protein, of 8480 M^-1^cm^-1^, at 280 nm measured in water, which corresponds to an absorbance of 0.715 at a concentration of 1 g/L. Regarding the amino acid composition, we find a high content of certain types of residues, which are expected to contribute to its folding pattern and functional properties. These residues include a relative high content of aliphatic and aromatic residues (12 L and 10 V, 4 A, 5 M, 4 F, 1 W, and 3 Y), which likely participate in creating the hydrophobic moment that is necessary for proteins to fold[14], as well as forming the aromatic cage, which is used by chromodomains to bind H3K9Me2 and H3K9Me3[15]. The presence of key basic residues, including 19 K and 4 R, is found in nuclear proteins, for which they form part of nuclear localization signals, as well as sites for acetylation, ubiquitination, and methylation; likely events involved in signaling within the cell nucleus. Phosphorylatable residues include 6 T, 2 S, and 2 Y. This data is in agreement with previous observations by our laboratory and others, which show that these residues are amenable to extensive modifications in HP1 proteins, working as a subcode in regulating the function of reader proteins that operate to fine-tune the histone code[16, 17]. The absence of the a CSD indicates that the protein is not able to form the expected CSD-to-CSD interactions that support homo- and hetero-dimerization of conventional HP1 proteins. Lastly, the protein has 13 E, which are known to be characteristic of CD-containing proteins likely for reinforcing their binding to histone tails that are in essence highly basic in nature[18]. Therefore, the new alternatively spliced mRNA, first identified from pancreatic cancer RNA-Seq data[12], as well as its predicted protein product would expand the number of HP1 isoforms in human tissues.

**Fig 2:**
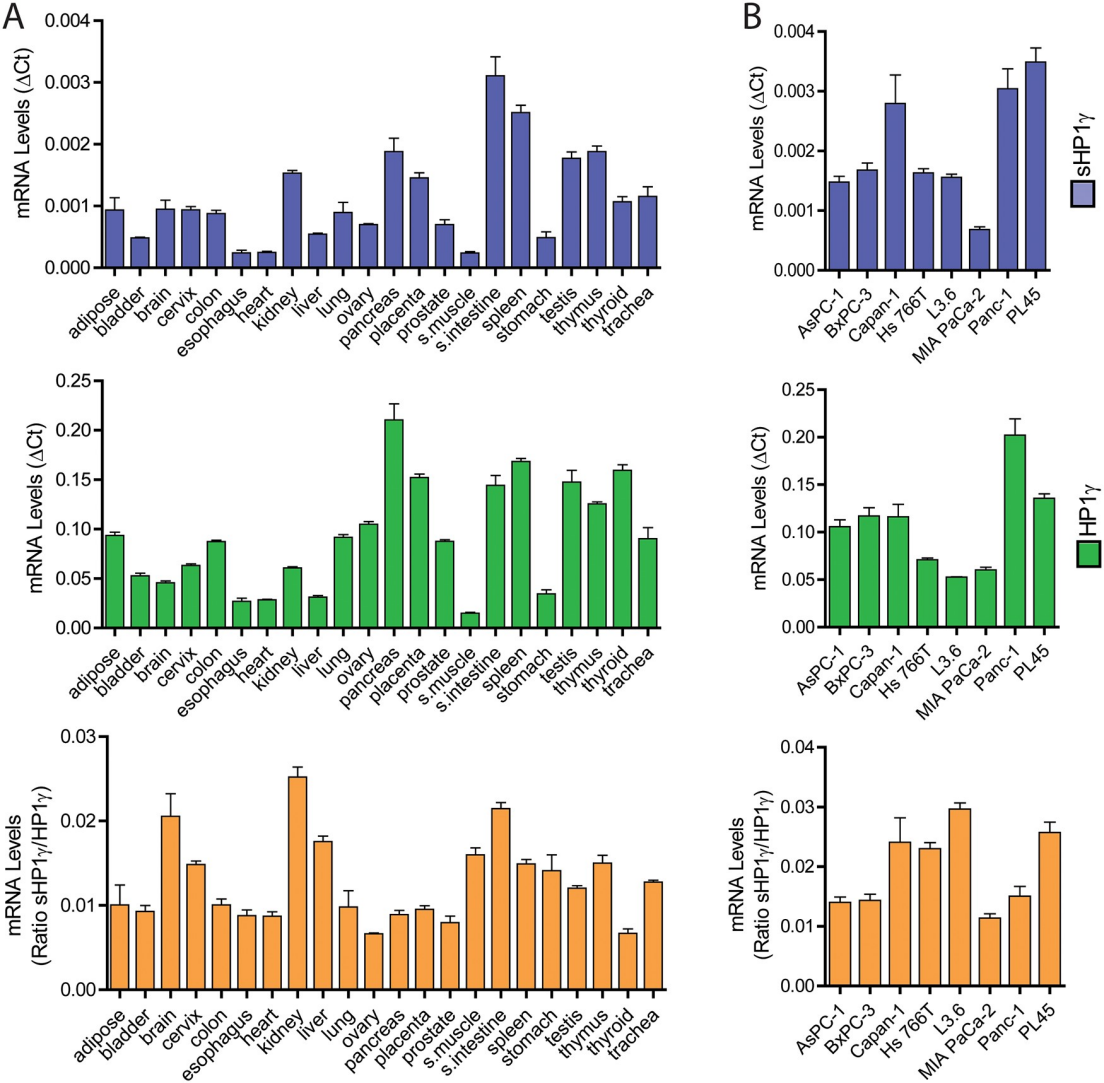
Detection of sHP1γ mRNA in human samples. (A) Isoform-specific qPCR assays were used to detect and compare the expression of the novel sHP1γ encoding mRNA (blue; top graph) with that of the long, conventional HP1γ isoforms (green; middle graph) in 22 different human tissues. The bottom graph (orange) represents the ratio of sHP1γ to HP1γ. (B) The same comparison between sHP1γ (blue; top graph) and the conventional HP1γ (green; middle graph) transcripts was performed in 8 pancreatic cancer cell lines. The bottom graph (orange) represents the ratio of sHP1γ to HP1γ.

Subsequently, we investigated the tissue distribution of sHP1γ mRNA. For this purpose, we first used an isoform-specific qPCR method, designed to specifically detect the two different splice junctions between exon 3 and 4 that are unique to the long and short HP1γ isoforms. Using this method, we compared the expression levels of the novel sHP1γ encoding mRNA with that of the conventional HP1γ isoform (Fig 2A), in a normal human tissue mRNA panel. The results of this experiment demonstrated that both types of mRNAs are expressed in a comparable pattern in most human tissues, however the overall expression of the short isoform is lower than the conventional HP1γ isoform yet present in all examined tissues. We found higher sHP1γ to HP1γ ratios of expression in the brain, cervix, kidney, liver, striated muscle, small intestine, spleen and thymus, and lowest ratios in ovary and thyroid. The same isoform comparison was made among 8 common pancreatic cancer cell lines (Fig 2B), in which we detect variable levels. Combined, these results demonstrate that the *CBX3* locus is spliced to give rise to a small isoform, sHP1γ, in various tissues.

**Fig 3:**
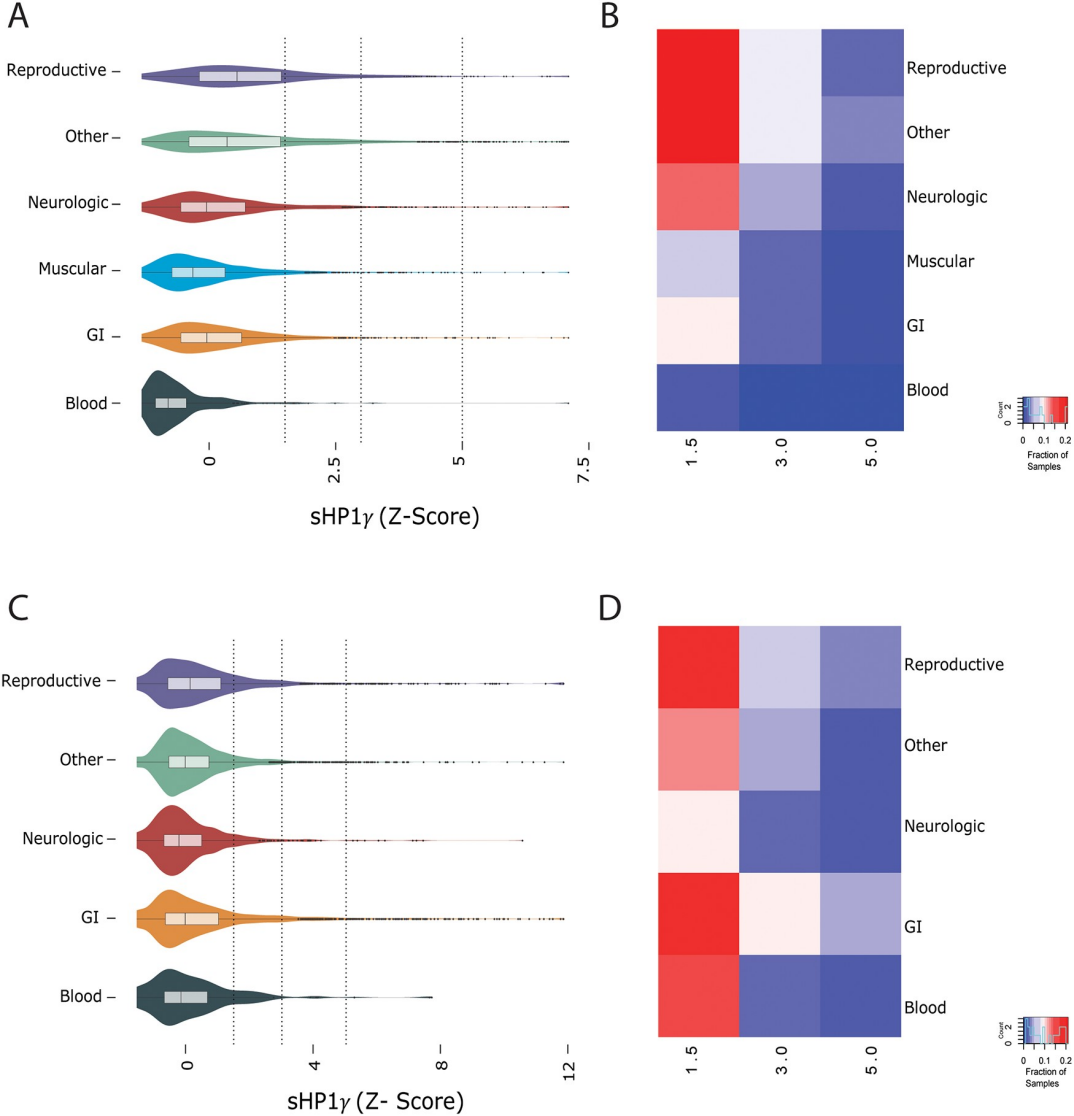
Expression level of sHP1γ across normal human and cancer tissues. Gene expression data gathered from GTEx shows the (A) distribution of gene expression levels across six groups of human tissues. Some tissues have markedly higher expression of sHP1γ than others. Vertical dotted lines indicate the global 85th, 95th and 97.5th percentiles. (B) The fraction of samples from each tissue group with an expression level at least the 85th (5 Transcripts Per Million—TPM), 95th and 97.5th percentiles are shown as a heatmap. Gene expression data extracted from TCGA shows the (C) distribution of gene expression levels across five groups of human tumors. Vertical dotted lines indicate the global 85th, 95th and 97.5th percentiles. (D) The fraction of samples from each tumor group with an expression level of at least the 85th (5 TPM), 95th and 97.5th percentiles are shown as a heatmap.

sHP1γ transcript expression in healthy adult human tissues was obtained by processing data derived from the Genotype-Tissue Expression project (GTEx)[19]. We analyzed 53 tissues subdivided in 6 major groups (Reproductive, Other, Neurologic, Muscular, Blood, and GI). We found that sHP1γ is present in all tissues (Fig 3A) at a lower level yet in a fairly consistent ratio to the conventional HP1γ isoform (S1 Fig). Long tails on these distributions indicate that there is a small number of normal human samples that have significant up-regulation of sHP1γ, with the heatmap further emphasizing these differences (Fig 3B). We also investigated sHP1γ expression in malignant tumors using data from the Cancer Genome Atlas (TCGA) (Fig 3C and 3D) with a particular focus on defining the ratio of short to long isoform for each cancer type (S2 Fig). We noted interesting variability in the ratio of short to long HP1γ among tumors, with the highest levels of sHP1γ being found in esophageal cancer (ESCA), ovarian cancer (OV) and stomach adenocarcinoma (STAD). Compared to non-tumor tissues, tumor groups are more uniform with a much higher fraction of tumor samples from GI tissues and the blood expressing a higher level of sHP1γ. Thus, these comparisons show that the two isoforms are expressed at a consistent ratio in normal human tissues, but this ratio becomes more variable in some types of cancer.

### sHP1γ is translated in human cells where it localizes to the nucleus

**Fig 4:**
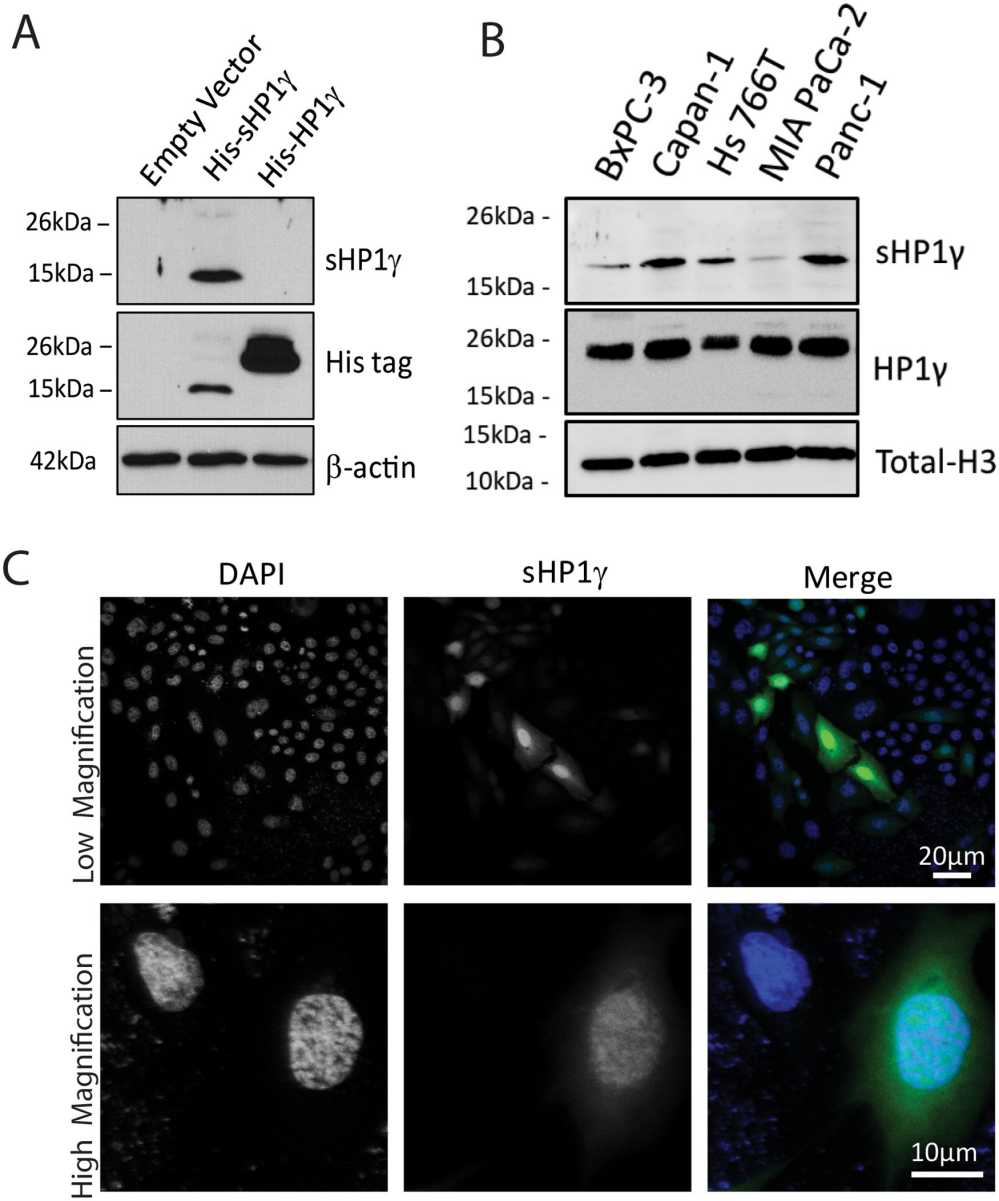
Detection of sHP1γ protein. (A) Lysates from CHO cells transfected with empty vector, His-sHP1γ and His-HP1γ were used for Western blot analyses with a newly generated peptide-specific antibody against sHP1γ. The overexpression of both HP1γ isoforms was confirmed with His antibody and β-actin was used as reference control. Molecular weight markers on the left illustrate the size difference of the two HP1γ isoforms. Full-length Western blot images are presented in S3A Fig (B) Lysates from five pancreatic cell lines were used for Western blot to analysis the relative expression of sHP1γ and HP1γ. Total H3 antibody was used as a loading control. Full-length Western blot images are presented in S3B Fig (C) Immunofluorescence analysis was performed for sHP1γ using our sHP1γ specific antibody (green) in HeLa cells. Independent fields of stained cells are shown at low and high magnification, upper and lower panel respectively. DAPI staining (blue) was carried out under optimum conditions to reveal nuclear structures.

Numerous alternatively spliced transcripts are often found in RNA-Seq studies; however, not all of these transcripts are reliably detected as alternative isoforms at the protein level[20]. To confirm that the transcript gives rise to a stable sHP1γ protein, we generated a polyclonal antibody against a synthetic peptide (RKEMLLTNQEDLPEVLILKE) for use in both, western blot (Fig 4A) and immunofluorescence (Fig 4B). The peptide was submitted to Basic Local Alignment Search Tool (BLAST, NCBI) and found to be present exclusively in the C-terminal region of sHP1γ. For the western blot analysis, we transiently transfected CHO cells with an epitope-tagged (His) form of sHP1γ or full length HP1γ, as a control, to induce expression of these isoforms. Fig 4A shows that the sHP1γ band is specifically detected in cells that overexpress the small isoform, and the antibody does not cross react with the full-length gene product. The overexpression of both isoforms was confirmed using a His tag antibody (Fig 4A). Five human pancreatic cancer cell lines were examined for their ability to express stable sHP1γ protein (Fig 4B). Capan-1 and Panc-1 showed higher expression of the short isoform when compared to Hs 766T, BxPC-3 and MIA PaCa-2. This data is consistent with the sHP1γ mRNA levels observed in Fig 2B. Therefore, this data demonstrates that the sHP1γ mRNA is expressed and translated into a stable protein *in vitro*.

Using an adenovirus construct and the same sHP1γ antibody, we transduced, fixed and conducted immunofluorescence confocal microscopy with HeLa cells to gain insight into the expression and localization of sHP1γ. In line with previous reports on the CSD-containing conventional HP1γ[21], we detected a strong signal for sHP1γ in the cell nucleus (Fig 4B), where other members of this family of chromatin proteins work. We also detected weak presence of sHP1γ in the cytoplasm, which may reflect the ability of these proteins to bind to α-importin, as demonstrated by our group, to transport HP1 proteins from the cytoplasm to the nucleus[8]. This experimental evidence is congruent with SLiM analyses, as performed by the ELM software[22], which identifies a monopartite NLS present in the non-conserved region of sHP1γ (a.a. 57–64). Interestingly, in contrast, the long canonical isoform bears a bipartite form of this motif that conforms to the KRKS-(X_9_)-KSKKKR consensus sequence. In spite of many years of investigations in the field of protein localization, no significant qualitative or quantitative differences have been reported between these two types of domains. Thus, functionally, they are both considered to be highly effective nuclear targeting sequences, which is congruent with our results that both isoforms primarily localize to the cell nucleus. In summary, the combined data from western blot and immunofluorescence analyses complement our findings with RNA-Seq and isoform-specific qPCR to reveal for the first time that the *CBX3/HP1γ* gene, which is regulated by alternative splicing, is translated to a shorter HP1γ protein in human cells where it primarily localizes to the cell nucleus. Thus, we subsequently studied the molecular properties of this novel HP1γ isoform, using sequence-based bioinformatics approaches, as well as homology-based molecular modeling and dynamic simulations.

### Modeling sHP1γ as a CSD-less protein with the ability to bind to methylated histone peptides

**Fig 5:**
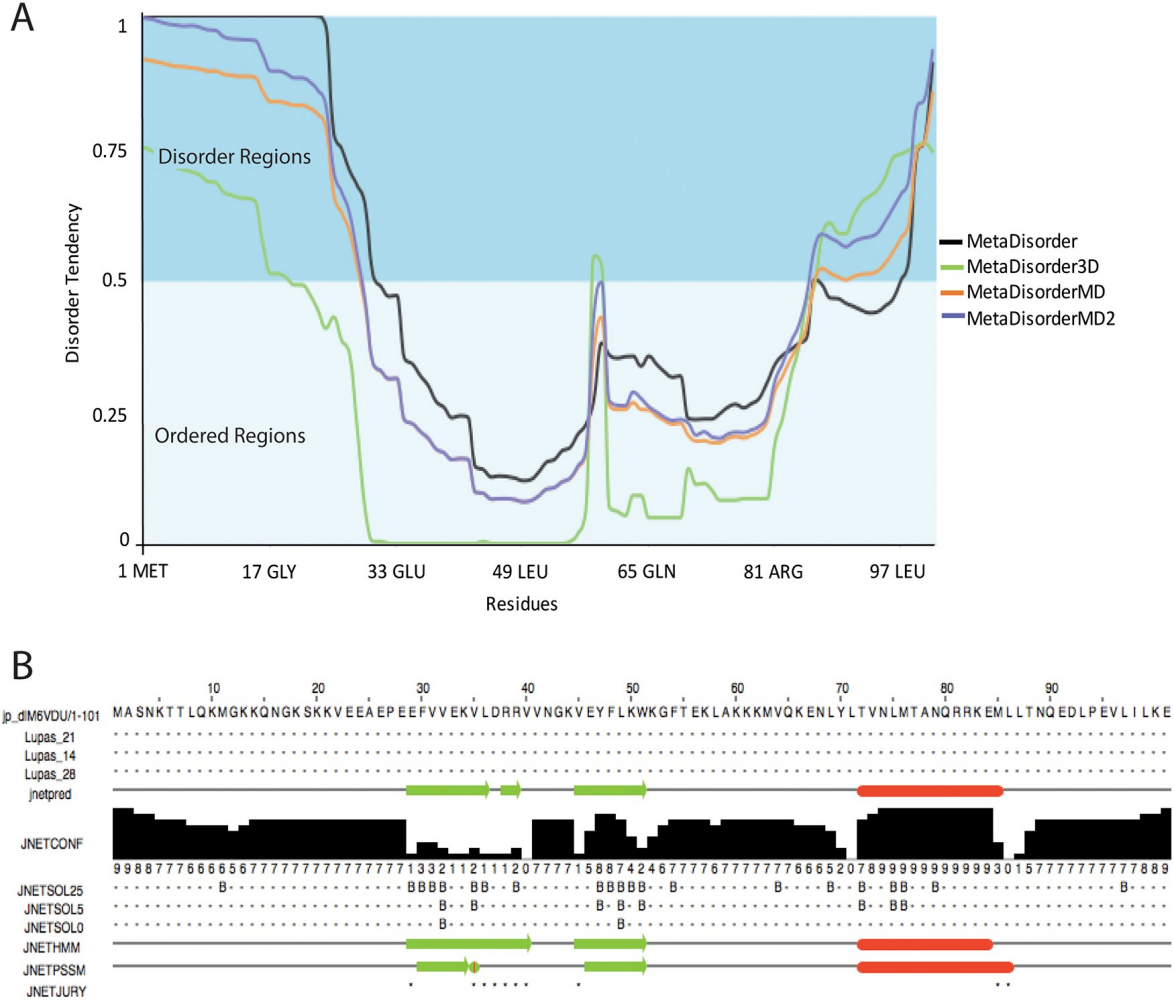
sHP1γ folding characteristics. (A) Protein disorder plot for sHP1γ generated by MetaDisorder software. All residues whose disorder probability is over 0.5 are considered as disordered. (B) The secondary structure of sHP1γ generated by JPRED4. β-sheets are marked as green arrows and α-helices as red bars.

To begin characterizing structural and molecular dynamic properties of this new sHP1γ protein, we utilized structural bioinformatics, molecular modelling, and molecular dynamic simulations. Order-to-disorder predictions indicated that both the N (1-Met to 30 -Phe) and C-terminal (97-Leu to 101-Glu) regions of sHP1γ are intrinsically disordered regions (IDR), while the residues located between them (31-Val to 96-Val) exhibit the opposite characteristics (Fig 5A). Interestingly, the final 45 amino acids at the C-terminus have no sequence similarities with other members of the CBX family, to which this protein belongs. However, fold recognition analyses using the JPRED4 algorithm[23] revealed that the sequence scores well for the CD protein fold and for protein folds with a similar organization, consisting of N-terminal β-sheets followed by an α-helix (Fig 5B). Critical assessment of structure predictions (CASP)-high scoring approaches including I-TASSER[24] and X-Raptor[25] used for sequence-to-structure predictions agree with this data (S4 Fig). Based on these analyses, the predicted organization of this protein is IDR-(β-sheets-α-helix)-IDR.

**Fig 6:**
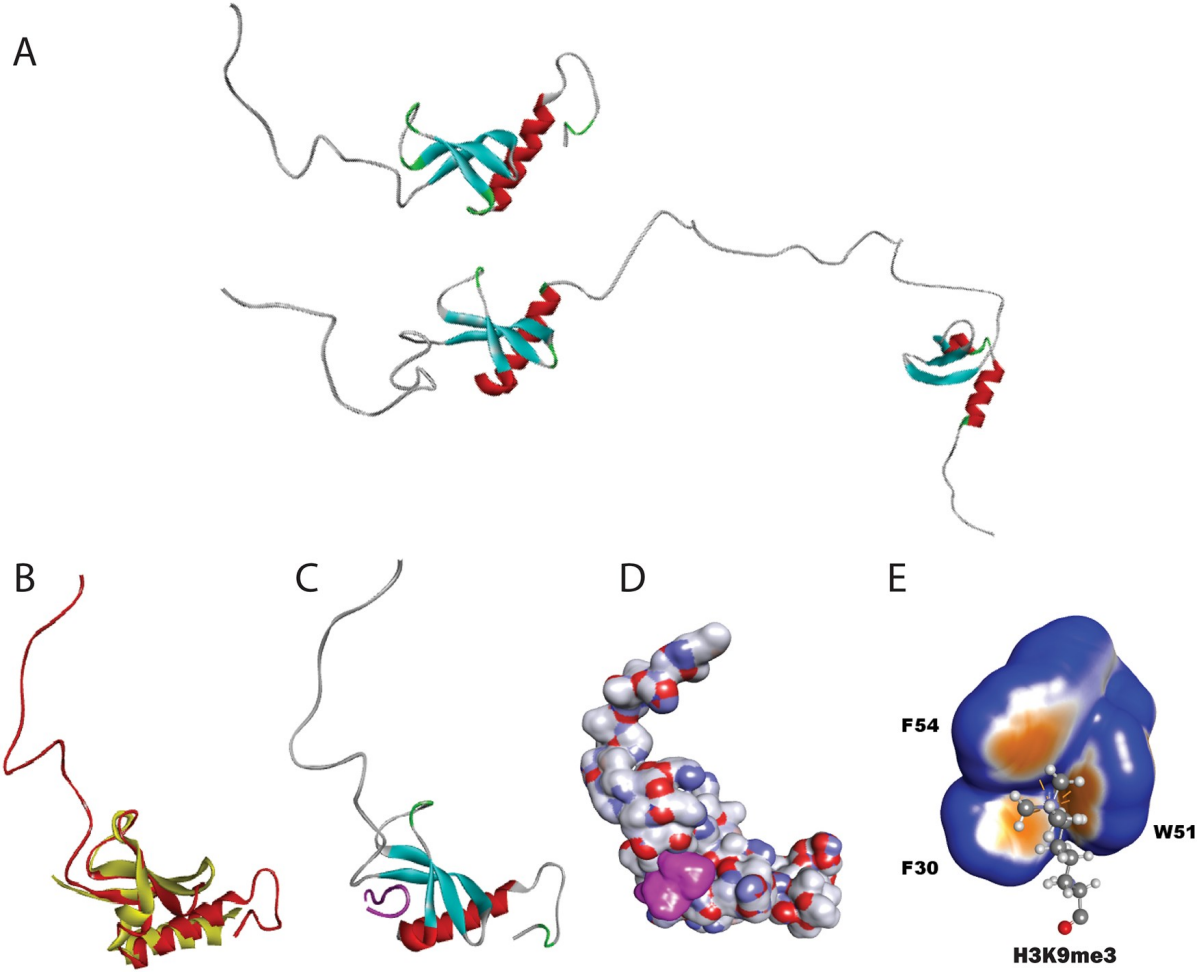
sHP1γ comparative and structural molecular modeling. (A) Molecular modeling of the novel sHP1γ (upper) and conventional full-length HP1γ (lower) isoforms in ribbon representation. The molecule can be divided into an N-terminal intrinsically disordered region (IDR; grey color), a chromodomain (β-sheets, light blue and α-helix, red), and a C-terminal IDR. (B) Structural model of sHP1γ (red) overlaid with MMP8 (yellow). (C) Structural model of sHP1γ complexed with a H3K9Me3 histone mark peptide (magenta). Tertiary structure showing how the chromoshadow-less sHP1γ accommodates a H3K9Me3 histone mark-containing peptide in a binding cavity provided by the chromodomain. The molecule can be divided into a N-terminal IDR (grey color), a Chromodomain (β-sheets colored light blue and α-helix, red). (D) Solvent accessible surface representation of sHP1γ (atom charge representation) reading the H3K9Me3 histone mark (magenta). Tertiary structure shows how the chromoshadow-less sHP1γ accommodates a H3K9Me3 histone mark-containing peptide in a binding cavity provided by the chromodomain. (E) Close view of the sHP1γ aromatic cage establishing contact with K3K9M3. The light brown areas correspond to more aromatic character.

**Fig 7:**
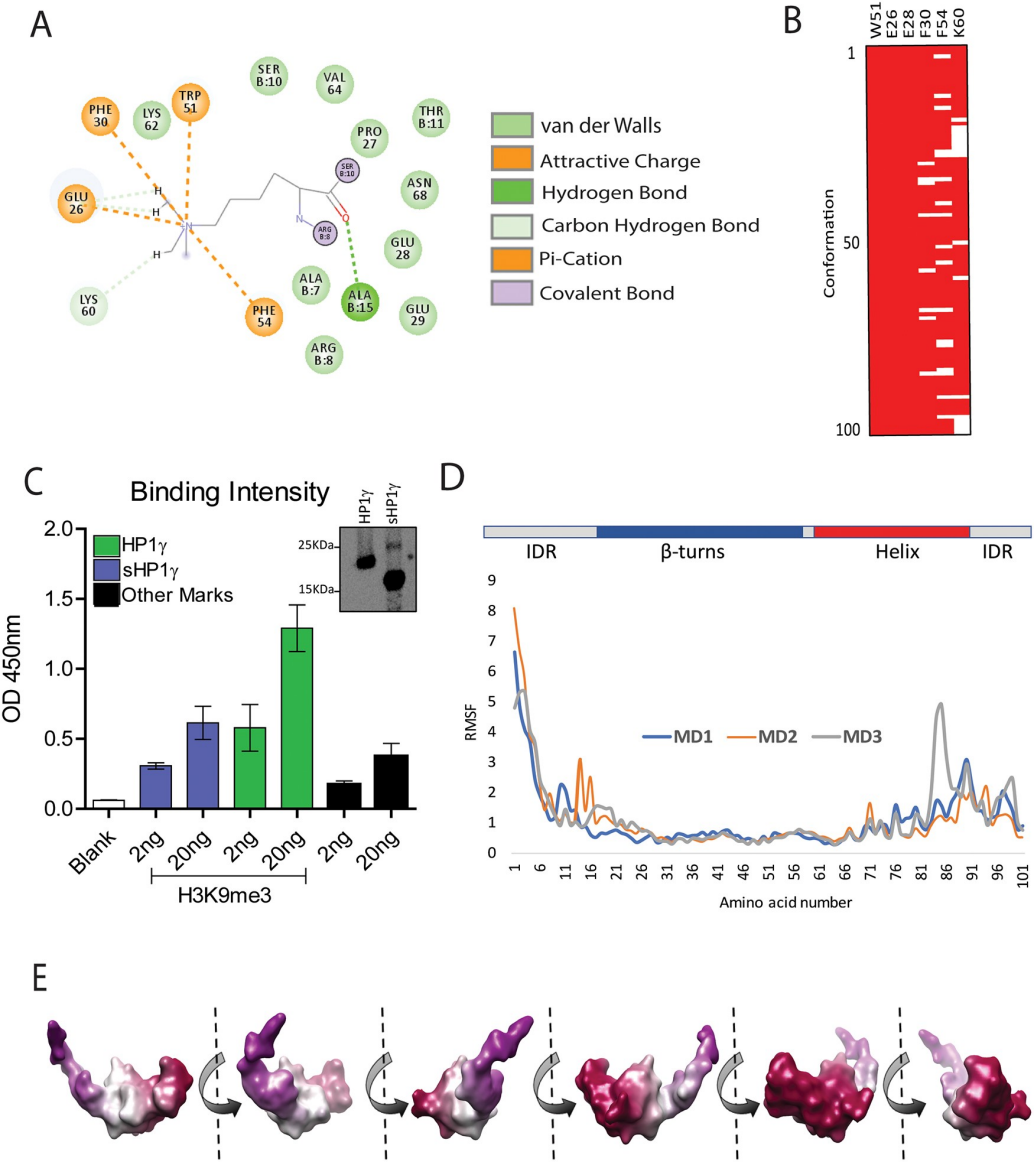
Time-dependent interaction of sHP1γ with the H3K9Me3 histone mark peptide. (A) 2D diagram of the bonding pattern of H3K9Me3 to the sHP1γ aromatic cage. The diagram also shows additional stabilizing bonds (A15, E26 and K60). (B) Binary representation of time-dependent interaction between sHP1γ and the H3K9Me3 peptide are shown with maintenance of contacts represented in red and loss of binding in white. Amino acids W51, E26, and E28 make contact with the H3K9Me3 residue in 100% of the conformations sampled during 5 nanosecond MD simulations. (C) Direct binding of sHP1γ to the H3K9Me3 histone mark. Purified sHP1γ and HP1γ proteins were tested for binding to histone H3 modifications by ELISA. The bar chart shows normalized values for the binding of sHP1γ (blue) and HP1γ (green) to two concentrations of H3K9Me3 mark. Black bars represent the average binding to other histone modifications. Error bars represent S.D. from duplicate independent experiments. Western blot (inset) shows purified sHP1γ and HP1γ proteins probed with an antibody to the N-terminus of HP1γ thereby recognizing both proteins simultaneously. Full-length Western blot images are presented in S3C Fig (D) The sHP1γ diagram shown at the top represents the secondary structural features of this protein. The low RMSF values corresponding to the β-turn containing regions (aromatic cage), which bind to the histone mark. (E) Surface-derived molecular properties of sHP1γ. Images correspond to all faces of which were used to determine the molecular properties listed on Table 3.

To shed light on the molecular properties of sHP1γ, we also generated a 3D model by satisfying spatial restraints deployed by MODELLER[26] and utilizing experimentally solved structures as templates (PDB IDs: 2L11, 3KUP, and 3DM1)[27, 28]. The structural model obtained for sHP1γ allows us to infer and compare characteristics of this isoform to the conventional isoform at atomic resolution and is shown in Fig 6A (sHP1γ upper, HP1γ lower). The quality of this model was high, with more than 96% of its residues being present in the allowed region of the Ramachandran plot (S5 Fig). Notably, this sHP1γ model reflects an N-terminal IDR followed by a globular domain containing the typical arrangement of an N-terminal three-stranded β-sheet that packs against a C-terminal α-helix, adjacent to a C-terminal IDR (Fig 6A). Within the conserved CD, the second and third β-strands can be connected by loops of variable lengths. However, CDs with longer or shorter linkers connecting the β-sheets to the helix are not found throughout evolution, as they would disrupt the core of structure[29]. This is consistent with the results of structural comparisons, which indicate that the CD of this human protein is conserved among isoforms and those present in evolutionarily distant organisms such as yeast, flies, and plants (Table 1). Along with these measurements of structural similarities, we show an example of a structural overlay between sHP1γ and MMP8 (Fig 6B), another CD-containing, CSD-less protein that binds to H3K9Me3, but is encoded by a non-HP1-encoding human locus[30]. Docking of the H3K9Me3 peptide to form a complex with sHP1γ was feasible through conservation of its CD with the CSD-containing conventional long HP1γ isoform (Fig 6C, 6D and 6E). Direct bonds formed between the aromatic cage of the sHP1γ CD and the H3K9Me3 mark are represented in Table 2 and S1 Table. The space between the turns and the helix defines a cavity or channel to horizontally accommodate the histone tail peptide (Fig 6C). A surface rendition of the sHP1γ-H3K9Me3 peptide complex, displayed in Fig 6D, better shows how the histone tail peptide becomes buried into this cavity. Figs 6E and 7A depict the contact between the H3K9Me3 mark and the aromatic cage formed by F30, W51, and F54. Molecular dynamics (MD) simulations (5 ns) revealed a time-dependent interaction between these aromatic residues and H3K9Me3 (Fig 7B). In addition, we noted during dynamic simulations that other residues, such as E26 and E28, are also critical for maintaining these intermolecular interactions (Fig 7B). To confirm that sHP1γ binds to H3K9Me3, purified histidine-tagged sHP1γ and HP1γ recombinant proteins were used to analyze their binding specificity to H3K9Me3 in an ELISA-based Histone H3 peptide array assay. Both proteins bound strongly, and an increase in binding was observed with the higher concentration of H3K9Me3 peptide (Fig 7C), demonstrating that sHP1γ indeed binds directly to the H3K9Me3 mark. Root mean square fluctuations (RMSF) values obtained during MD simulations, as a measure of regional displacements, demonstrated that the while the IDR and helix region of these protein are quite dynamic, binding to the histone tail restricts the fluctuations of the CD (Fig 7D). MD simulations comparing the peptide bound form (holo) to the non-peptide bound form (apo) of sHP1γ (S6 Fig) demonstrates that in the absence of the peptide the chromobox domain of the small HP1 displays higher RMSF values when compared with the protein bound to the peptide. This result indicates that like in many other proteins, binding to its target stabilizes the complex showing less dynamic range of motion. To guide protein purification experiments that consider hydrodynamic radius, such as in gel filtration or ultracentrifugation, we modeled the surface properties of sHP1γ as a globular protein in solution (Fig 7E). We measured the molecular properties of this protein when given a water-accessible surface, according to the method of Neil R. Voss and Mark Gerstein[31]. These properties, which are listed in Table 3, when compared to the conventional HP1γ as modeled by Velez, *et al*.[8] indicate that sHP1γ is significantly smaller (sHP1γ volume 16443Å^3^ as compared to HP1γ, 32517Å^3^) and more spherical (sHP1γ 0.53Ψ as compared to HP1γ 0.39Ψ). Therefore, although shorter in length and different in sequence, sHP1γ appears to conserve some of the most salient structural features of the CBX family of proteins. These findings should be taken into consideration when studying the repertoire of HP1 proteins expressed in humans.

**Table 1:** Similarities of sHP1γ to chromodomain containing proteins across kingdoms. Root mean square deviation (RMSD) values provide insight into the similarities of sHP1γ to organisms ranging from human to *saccharomyces pombe* (1e0b-B) and *drosophilae melanogaster* (1kne-A, and 5xyw-B) to even plants (4iut-A). The protein data bank (PDB) identifiers are listed for each ortholog.

PROTEIN	PDB	RMSD
CBX3	2l11-A	0.7
CBX5	3fdt-A	1
CBX6	3i90-A	1.1
CBX7	4mn3-A	1.2
*d*.*m* HP1	1kne-A	1.2
RHINO	5xyw-B	1.2
*d*.*m* PLC	1pdq-A	1.3
MMP8	3r93-C	1.4
SUV39H1	3mts-C	1.4
CBX8	3i91-A	1.4
CBX2	5epk-A	1.5
a.t. SAWADEE	4iut-A	1.5
CBX1	3g7l-A	1.6
*s*.*p*. HP1	1e0b-B	1.7
CDYL-2	5jjz-A	1.7

**Table 2:** Direct bonds formed between the aromatic cage of sHP1γ CD and the H3K9me3 mark. Distance cut-offs for electrostatic and hydrogen bonds were 5 and 3, respectively.

Bond Type	From Residues	To Residues
**Hydrogen Bond**	C:M3L9:HN	A:GLU39:0
**Hydrogen Bond**	C:M3L9:HM31	A:PHE42
**Electrostatic**	C:M3L9:NZ	A:PHE42
**Electrostatic**	C:M3L9:NZ	A:TRP65
**Electrostatic**	C:M3L9:NZ	A:TRP65

**Table 3:** Surface-derived molecular properties of sHP1γ.

Property	Unit
**Voxel Size**	0.5 Å
**Volume**	16443 Å^3^
**Surface Area**	5942 Å^2^
**Sphericity**	0.53 Ψ
**Effective Radius**	8.30 Å
**Center of Mass**	(-13.0, -4.7, 0.9) Å

## Discussion

HP1 was one of the first epigenomic regulators identified through its function in heterochromatin formation. Thus, the current study contributes significantly to advance this field, by reporting the identification and characterization of a small HP1γ isoform. The novel isoform was discovered as a result of our long-term efforts to better understand the role of this protein family in pancreatic cancer[32, 33]. Reanalysis of RNA-Seq samples from pancreatic cancer cells[12] led us to define that the *CBX3* locus gives rise to mRNA that encodes a novel spliced form of the *CBX3* locus, which we termed small HP1γ, sHP1γ. We present both bioinformatics-based and experimentally-derived evidence demonstrating that the mRNA for this novel isoform is widely expressed in both normal and many different cancer types of human tissues. A peptide-specific antibody against sHP1γ confirmed that this protein is indeed translated. Furthermore, immunofluorescence-based microscopy corroborated bioinformatics-based predictions for this protein with localization to the cell nucleus. Thus, combined, these experiments report, for the first time, the existence of a *bona fide* CSD-less HP1γ isoform, which is not specific to PDAC, but rather widely expressed in a variety of human tissues.

To gain insight into the molecular properties of this novel isoform at an atomic level, we modeled the structure and dynamics of this protein, using similar methodologies to those we have previously applied to study properties of the full-length conventional HP1γ isoform[8]. Sequence analyses demonstrated that sHP1γ shares complete conservation of the CD region, which contains the residues that form the aromatic cage used by HP1 and Polycomb (Pc) CBX family members for binding methylated histone 3 at K9 and K27, respectively. Notably, the rest of the protein does not contain homology to the larger isoforms. A meta, order-to-disorder analysis of the CD predicted that sHP1γ will likely fold in a manner that preserves the methylated histone binding function of this domain. Structural predictions that use a combination of threading (as in Fig 5B) and homology-based (as in Fig 6) methods revealed the potential of sHP1γ to adopt a structure that is highly similar to most members of the HP1/Pc CBX family of proteins[28]. Pure homology modeling by satisfaction of special restraints using MODELLER[26] was congruent with the other methods. To gain insight into the molecular properties of this protein, we built a model of sHP1γ both, in its free form and when bound to the H3K9Me3 peptide. Molecular dynamic simulations suggested that the properties of this protein are consistent with those previously described for other family members (HP1α, HP1β, HP1γ)[8]. When compared with the conventional HP1γ isoform, however, this protein lacks a C-terminal CSD domain. On the other hand, the structure of this protein highly resembles another H3K9Me3 binding protein, MPP8, which also lacks a CSD and is encoded by a gene distinct from *CBX3*[30]. Thus, our study provides evidence that an expanded number of proteins can bind to H3K9Me3. These proteins can be divided into two groups: those proteins that contain a C-terminal CSD and another set of proteins which lack this domain. From this observation, we can draw inferences that are important to consider as it relates to sHP1γ. For instance, the lack of the CSD used for heterodimerization among the conventional HP1α, HP1β, and HP1γ, as well as for recruiting many other partners that regulate many cellular functions, is highly suggestive that sHP1γ will have some divergent functions. If the protein behaves more like other CSD-less CBX members[29], one could expect that it may use the CD for the dual function of binding histones and forming complexes with other proteins. However, the formation of complexes with other HP1-interacting proteins may not be feasible for sHP1γ. For example, the conventional HP1γ isoform recruits its histone methyl transferase partner, SUV39H1, through its dimerized CSD. The dimerized CSD forms a nonpolar groove that can accommodate penta-peptides with the consensus sequence motif PXVXL, found in many HP1-interacting proteins, including SUV39H1[4]. Thus, it is not expected that sHP1γ would be able to perform this function, at least through a similar binding mechanism, due to its lack of a CSD. On the other hand, G9a/EHMT2 is another histone methyl transferase that is recruited by HP1γ through its CD[34], a phenomenon in which sHP1γ may also participate. Thus, we are optimistic that future studies focused on directly addressing this question will further contribute to define the complexity of this system.

Notably, although the scope and value of this study is that it extends the knowledge of HP1 proteins by characterizing the first human alternative spliced chromoshadow-less isoform at the molecular level, we provide additional information as to its localization, expression, and histone reading properties. Consequently, we believe that this information must be taken into consideration by additional researchers in the field, who only associate the function of HP1 protein to the longer isoforms. This is important since the failure to recognize its existence may impact negatively on the interpretation of future ChIP-Seq and similar studies. However, additional investigations are still necessary to gain insight into its genome-wide distribution and the cell biological function of this protein as it relates to cancer initiation, progression, differentiation, cell cycle control, DNA repair, chromosomal stability, senescence, among others, which are functions previously described by the combination of a large number of studies[35–60]. Thus, while gaining information on these properties is important, the extent and nature of these analyses require several, carefully designed experimentations, which thereby fall outside of the scope of the current report. We are, however, optimistic that future experiments from our laboratory and others will further illuminate whether and how this protein participates in these processes.

In conclusion, we have used a combination of experimental, bioinformatics, modeling, and molecular dynamics methods in the current study to describe the existence of a novel CSD-less sHP1γ. This discovery extends the repertoire of proteins that may bind H3K9Me3 under similar or distinct functional contexts. Because of the known role of these proteins in both physiological and pathological processes, this data bears both biological and biomedical relevance. Furthermore, the insight provided here offers speculation on the regulation of pathways utilizing HP1 proteins and broadens the functional repertoire of these important epigenomic regulators for future investigations.

## Materials and methods

### Cell lines and reagents

Human pancreatic cells, CHO and HeLa cells were obtained from American Type Culture Collection (ATCC) and cultured in appropriate media at 37 °C with 5% CO_2_ according to recommendations. Bruns *et al*.[61] originally isolated the L3.6 cells which were maintained in minimum essential media (Invitrogen) supplemented with 10% fetal bovine serum, 2mM L-glutamine (Gibco), 1x MEM nonessential amino acids (Gibco), 2x MEM vitamins (Gibco), 1mM sodium pyruvate (Gibco) and 0.1% antibiotic/antimycotic (Invitrogen). Standard molecular biology techniques were used to clone sHP1γ and HP1γ, as previously described[44] into pcDNA3.1-His and Ad5CMV vectors. For transient expression in CHO cells, 1x10^6 cells were plated in 60mm dishes and allowed to attach overnight. Cells were allowed to recover for 48 hours after Lipofectamine2000 (Invitrogen) transfection with 8μg of the His-HP1γ, His-sHP1γ or His-Empty Vector control plasmid. Recombinant adenovirus for sHP1γ and HP1γ[44] was generated through the Gene Transfer Vector Core at the University of Iowa, IA, USA. The generation and purification of an antibody against sHP1γ was completed using a similar method as previously described by our laboratory for the canonical isoform[16] using the immunogen peptide with the RKEMLLTNQEDLPEVLILKE primary sequence.

### Identification and tissue distribution of the sHP1γ encoding mRNA

Evaluation of the normal human tissue panel was performed using FirstChoice^®^ Human total RNA Survey Panel following manufacturer’s recommendations. Total RNA for stomach and pancreas tissues were purchased separately from Agilent/Stratagene (Catalog# 540023 pancreas and 5400037 stomach). For human pancreatic cancer cell lines, total RNA was isolated with the Qiagen RNeasy kit according to the manufacturer’s protocol. RNA was reverse-transcribed using the SuperScript III System (Invitrogen), and SYBR Green-based real-time PCR was performed according to the manufacturer’s instructions (Bio-Rad). Primers for isoform-specific qPCR were designed with the following sequences: sHP1γ F: 5-GTGGAAGGGATTTACAGAAAAGC-3, sHP1γ R: 5-TTTGCCAGAGGTCTTGATCC-3, HP1γ F: 5-AAGGGATTTACAGATGCTGAC-3 and HP1γ R: GACAAACCAAGAGGATTTGC.

### Analysis of isoform expression across publicly available data

Normalized gene expression data were gathered from the GTEx and TCGA consortia. Isoform expression from TCGA was downloaded from TSVdb. Because these consortia distribute data using slightly different normalization methods (GTEx distributes TMM and TSVdb RPKM values), we standardized gene expression within each study using z-score calculated as the per-gene median divided by the per-gene median absolute value.

### Western blot analysis

For Western blots, transfected CHO cells were lysed in 4× Laemmli buffer (250 mm Tris (pH 6.8), 20% glycerol, 8% SDS, 0.0025% bromophenol blue, 1 mm *β*‐mercaptoethanol), collected by scraping with a rubber policeman, sonicated for 10s, and boiled at 95 °C for 5 min prior to loading. Lysates were subjected to 14% SDS polyacrylamide gel electrophoresis, transferred onto polyvinylidene difluoride membranes and probed with primary antibodies: anti‐ sHP1γ (1:5000), HP1γ (1:1000, abcam) or His-tag Omni-probe (D-8) (1:500, Santa Cruz Biotechnology) diluted in 5% milk in tris buffered saline with tween (TBST). Anti‐rabbit secondary antibodies (Millipore) were incubated on the membranes for 1 h at room temperature, after three successive washes with TBST, bands were detected with enhanced chemiluminescence (ECL, Pierce).

### Immunofluorescence

HeLa cells were plated on poly-L-lysine‐coated circular coverslips (0.1 mg/mL poly-L-lysine) and allowed to adhere overnight. Transduction occurred at a multiplicity of infection of 400:1. After 48 h, cells were fixed with 4% formaldehyde, permeabilized with 0.2% Triton‐X 100, blocked for 30 min, and finally stained with the anti-sHP1γ antibody (1:250) and Alexa Fluor 488 anti-rabbit secondary antibody (1:500, Invitrogen). Coverslips were mounted in VectaShield mounting media with DAPI for immunofluorescence. Images were acquired using 40× and 100× objective lenses on a Zeiss LSM 780 confocal microscope.

### Protein purification and modified histone H3 binding assay

sHP1γ was codon optimized for expression in *E*. *coli* and chemically synthesized by Genescript in the pET15b vector. Full length HP1γ cDNA was cloned into the pET21b vector as well, to generate plasmids which express the proteins fused to an N-terminal His-tagged. Fusion protein expression was induced in BL21 (DE3) cells by induction at an OD_600_ of 0.4 with 0.1mM isopropyl-β-D-thiogalactopyranoside (IPTG) and incubation for an additional 2 hours at 32 °C. Cells were lysed and protein subsequently purified by using HisPur^™^ Ni-NTA columns (ThermoFisher) in accordance with the manufacturer’s instructions. H3K9Me3 binding specificity was detected using the Pre-Sure^™^ Histone H3 Peptide Array ELISA Kit (Epigentek) with small modifications from the manufacturer’s protocol. Purified sHP1γ and HP1γ histidine-tagged proteins were diluted to a concentration of 1ug/ml, added to the array plate and incubated for 2 hours at room temperature. His-tag Omni-probe (D-8) antibody was diluted to 0.4ug/ml and used as primary antibody to detect the binding of sHP1γ and HP1γ to histone peptides. Arbitrary units were calculated based on the absorbance (450 nm) to represent the relative levels of binding specificity.

### Molecular modeling and molecular dynamic simulations

The modeling for the sHP1γ alone and in complex with the H3K9Me3 peptide was performed using a combination of approaches previously described[8]. Briefly, homology-based modeling was performed using MODELLER[26] with previously solved structures as templates (PDB IDs: 2L11, 3KUP, and 3DM1)[27, 28]. The model was refined by energy minimization using two cycles of 200 steps of steepest descent and two cycles of Random Newton before proper evaluation of stereochemical properties by the Ramachandran method. Molecular dynamic simulations were performed for 5 nanoseconds, each using different random seeds, an isothermal-isobaric (NPT) ensemble, and the distance-dependent dielectric method for simulating implicit solvent conditions. Bonds were modelled by a distance dependence approach. Since it is known that the chromodomain-H3K9Me3 complex forms by an induced fitting mechanism, a soft harmonic restraint, using the best fit intermolecular interaction method, was applied to the structure. Visualization and illustration were done using Discovery Studio (Biova).

## Supporting information

S1 Table Additional bonds that further stabilize sHP1γ—Histone 3 tail complex. (DOCX)

S1 Fig sHP1γ has variable expression across normal human tissues. Using the GTEx dataset, we show the distribution of gene expression for the sHP1**γ** isoform, for each body tissue and using smoothed density (violin) plots. The protein is expressed at a low level in most human tissues. Tissues are colored tan for brain regions, dark red for primary gastrointestinal track, red for arterial and cardiac tissues, and pink for all others.
(DOCX)

S2 Fig sHP1γ is highly expressed in a subset of nearly all tumors. Using TCGA data, we show the ratio of short to long isoform for each cancer type, designating each cancer type by its official abbreviation. A horizontal line marks the median value across all samples. Cancer types are colored by their median value. Many cancer types have the majority of their samples below the global median, while others have many samples above the global median, demonstrating that the two isoforms are likely regulated in different ways by cancers of different tissues.
(DOCX)

S3 Fig Full length Western blot images from Figs 4A, 4B and 7C. (A) Lysates from CHO cells transfected with empty vector, His-sHP1γ and His-HP1γ were used for Western blot analyses with a newly generated peptide-specific antibody against sHP1γ. The overexpression of both HP1γ isoforms was confirmed with His antibody and β-actin was used as reference control. Molecular weight markers on the left illustrate the size difference of the two HP1γ isoforms. The cropped images for lanes 5–7 shown in Fig 4A. Lanes 1–4 and 8–9 were samples for an irrelevant study. (B) Lysates from five pancreatic cell lines were used for Western blot to analysis the relative expression of sHP1γ and HP1γ. Total H3 antibody was used as a loading control. The cropped images are presented in Fig 4B. (C) Western blot of purified sHP1γ and HP1γ proteins probed with an antibody to the N-terminus of HP1γ to recognize both proteins, simultaneously. The cropped images are shown in Fig 7C.
(DOCX)

S4 Fig Sequence-to-structure prediction by high CASP performer algorithms. (A) 3D structure of sHP1γ as modelled by I-TASSER, (B) 3D structure of sHP1γ generated using X-Raptor. Note that both are remarkably similar to each other and to the homology-based model depicted in Fig 6A.
(DOCX)

S5 Fig Ramachandran plot of sHP1γ. The quality of the structural model is high in that 96% of its residues are present in the expected region.
(DOCX)

S6 Fig RMSF values of H3K9Me3-bound and unbound sHP1γ. Molecular dynamics (MD) simulations (5 ns), shown in duplicate, comparing the peptide bound form (Holo) to the non-peptide bound form (Apo) of sHP1γ. Note that binding to the H3K9Me3 histone mark-containing peptide stabilizes the complex and reduces the intrinsic flexibility of the chromodomain.
(DOCX)

## Abbreviations

CBX3: Chromobox containing protein 3
CD: chromodomain
CSD: chromoshadow domain
HP1: Heterochromatin protein 1
IDR: intrinsically disorder region

## References

[1] Eissenberg JC, James TC, Foster-Hartnett DM, Hartnett T, Ngan V, Elgin SC. Mutation in a heterochromatin-specific chromosomal protein is associated with suppression of position-effect variegation in Drosophila melanogaster. Proceedings of the National Academy of Sciences of the United States of America. 1990;87(24):9923–7. 10.1073/pnas.87.24.9923 . 2124708 PMC55286

[2] James TC, Elgin SC. Identification of a nonhistone chromosomal protein associated with heterochromatin in Drosophila melanogaster and its gene. Molecular and cellular biology. 1986;6(11):3862–72. 10.1128/mcb.6.11.3862 . 3099166 PMC367149

[3] Vermaak D, Henikoff S, Malik HS. Positive selection drives the evolution of rhino, a member of the heterochromatin protein 1 family in Drosophila. PLoS genetics. 2005;1(1):96–108. Epub 2005/07/25. 10.1371/journal.pgen.0010009 . 16103923 PMC1183528

[4] Lomberk G, Wallrath L, Urrutia R. The Heterochromatin Protein 1 family. Genome biology. 2006;7(7):228-. Epub 2006/07/21. 10.1186/gb-2006-7-7-228 . 17224041 PMC1779566

[5] Bannister AJ, Zegerman P, Partridge JF, Miska EA, Thomas JO, Allshire RC, et al Selective recognition of methylated lysine 9 on histone H3 by the HP1 chromo domain. Nature. 2001;410:120 10.1038/35065138 11242054

[6] Lachner M, O’Carroll D, Rea S, Mechtler K, Jenuwein T. Methylation of histone H3 lysine 9 creates a binding site for HP1 proteins. Nature. 2001;410:116 10.1038/35065132 11242053

[7] Fanti L, Pimpinelli S. HP1: a functionally multifaceted protein. Current Opinion in Genetics & Development. 2008;18(2):169–74. 10.1016/j.gde.2008.01.009 18329871

[8] Velez G, Lin M, Christensen T, Faubion WA, Lomberk G, Urrutia R. Evidence supporting a critical contribution of intrinsically disordered regions to the biochemical behavior of full-length human HP1γ. Journal of molecular modeling. 2016;22(1):12-. Epub 2015/12/17. 10.1007/s00894-015-2874-z . 26680990 PMC4683166

[9] Dialynas GK, Vitalini MW, Wallrath LL. Linking Heterochromatin Protein 1 (HP1) to cancer progression. Mutation research. 2008;647(1–2):13–20. Epub 2008/09/24. 10.1016/j.mrfmmm.2008.09.007 . 18926834 PMC2637788

[10] Jones DO, Cowell IG, Singh PB. Mammalian chromodomain proteins: their role in genome organisation and expression. BioEssays. 2000;22(2):124–37. 10.1002/(SICI)1521-1878(200002)22:2<124::AID-BIES4>3.0.CO;2-E 10655032

[11] Velez G, Urrutia R, Lomberk G. Critical Role of the HP1-Histone Methyltransferase Pathways in Cancer Epigenetics. Medical Epigenetics. 2013;1(1):100–5. 10.1159/000355978

[12] Lomberk G, Blum Y, Nicolle R, Nair A, Gaonkar KS, Marisa L, et al Distinct epigenetic landscapes underlie the pathobiology of pancreatic cancer subtypes. Nature Communications. 2018;9:1978 10.1038/s41467-018-04383-6 . 29773832 PMC5958058

[13] Gasteiger E, Hoogland C, Gattiker A, Duvaud Se, Wilkins MR, Appel RD, et al Protein Identification and Analysis Tools on the ExPASy Server In: Walker JM, editor. The Proteomics Protocols Handbook. Totowa, NJ: Humana Press; 2005 p. 571–607.

[14] Silverman BD. Hydrophobic moments of protein structures: Spatially profiling the distribution. Proceedings of the National Academy of Sciences. 2001;98(9):4996 10.1073/pnas.081086198 11309489 PMC33152

[15] Yap KL, Zhou M-M. Keeping it in the family: diverse histone recognition by conserved structural folds. Critical reviews in biochemistry and molecular biology. 2010;45(6):488–505. Epub 2010/10/06. 10.3109/10409238.2010.512001 . 20923397 PMC2988946

[16] Lomberk G, Bensi D, Fernandez-Zapico ME, Urrutia R. Evidence for the existence of an HP1-mediated subcode within the histone code. Nature Cell Biology. 2006;8:407 10.1038/ncb1383 16531993

[17] LeRoy G, Weston JT, Zee BM, Young NL, Plazas-Mayorca MD, Garcia BA. Heterochromatin protein 1 is extensively decorated with histone code-like post-translational modifications. Molecular & cellular proteomics: MCP. 2009;8(11):2432–42. Epub 2009/06/30. 10.1074/mcp.M900160-MCP200 . 19567367 PMC2773712

[18] Wu S-J, Tambyraja R, Zhang W, Zahn S, Godillot AP, Chaiken I. Epitope Randomization Redefines the Functional Role of Glutamic Acid 110 in Interleukin-5 Receptor Activation. Journal of Biological Chemistry. 2000;275(10):7351–8. 10.1074/jbc.275.10.7351 10702307

[19] Consortium GT. The Genotype-Tissue Expression (GTEx) project. Nature genetics. 2013;45(6):580–5. 10.1038/ng.2653 . 23715323 PMC4010069

[20] Tress ML, Abascal F, Valencia A. Alternative Splicing May Not Be the Key to Proteome Complexity. Trends in Biochemical Sciences. 2017;42(2):98–110. 10.1016/j.tibs.2016.08.008 27712956 PMC6526280

[21] Charó NL, Galigniana NM, Piwien-Pilipuk G. Heterochromatin protein (HP)1γ is not only in the nucleus but also in the cytoplasm interacting with actin in both cell compartments. Biochimica et Biophysica Acta (BBA)—Molecular Cell Research. 2018;1865(2):432–43. 10.1016/j.bbamcr.2017.11.015 29208528

[22] Gouw M, Michael S, Sámano-Sánchez H, Kumar M, Zeke A, Lang B, et al The eukaryotic linear motif resource—2018 update. Nucleic acids research. 2018;46(D1):D428–D34. 10.1093/nar/gkx1077 . 29136216 PMC5753338

[23] Drozdetskiy A, Cole C, Procter J, Barton GJ. JPred4: a protein secondary structure prediction server. Nucleic acids research. 2015;43(W1):W389–W94. Epub 2015/04/16. 10.1093/nar/gkv332 . 25883141 PMC4489285

[24] Zhang Y. I-TASSER server for protein 3D structure prediction. BMC Bioinformatics. 2008;9:40-. 10.1186/1471-2105-9-40 . 18215316 PMC2245901

[25] Källberg M, Wang H, Wang S, Peng J, Wang Z, Lu H, et al Template-based protein structure modeling using the RaptorX web server. Nat Protoc. 2012;7(8):1511–22. 10.1038/nprot.2012.085 . 22814390 PMC4730388

[26] Šali A, Blundell TL. Comparative Protein Modelling by Satisfaction of Spatial Restraints. Journal of Molecular Biology. 1993;234(3):779–815. 10.1006/jmbi.1993.1626 8254673

[27] Ruan J, Ouyang H, Amaya MF, Ravichandran M, Loppnau P, Min J, et al Structural basis of the chromodomain of Cbx3 bound to methylated peptides from histone h1 and G9a. PloS one. 2012;7(4):e35376–e. 10.1371/journal.pone.0035376 . 22514736 PMC3325965

[28] Kaustov L, Ouyang H, Amaya M, Lemak A, Nady N, Duan S, et al Recognition and specificity determinants of the human cbx chromodomains. The Journal of biological chemistry. 2011;286(1):521–9. Epub 2010/11/03. 10.1074/jbc.M110.191411 . 21047797 PMC3013012

[29] Nielsen PR, Callaghan J, Murzin AG, Murzina NV, Laue ED. Expression, Purification, and Biophysical Studies of Chromodomain Proteins Methods in Enzymology. 376: Academic Press; 2003 p. 148–70. 10.1016/S0076-6879(03)76010-8 14975304

[30] Kokura K, Sun L, Bedford MT, Fang J. Methyl-H3K9-binding protein MPP8 mediates E-cadherin gene silencing and promotes tumour cell motility and invasion. The EMBO journal. 2010;29(21):3673–87. Epub 2010/09/24. 10.1038/emboj.2010.239 . 20871592 PMC2982762

[31] Voss NR, Gerstein M. 3V: cavity, channel and cleft volume calculator and extractor. Nucleic acids research. 2010;38(Web Server issue):W555–W62. Epub 2010/05/16. 10.1093/nar/gkq395 . 20478824 PMC2896178

[32] Lomberk GA, Iovanna J, Urrutia R. The promise of epigenomic therapeutics in pancreatic cancer. Epigenomics. 2016;8(6):831–42. Epub 2016/06/23. 10.2217/epi-2015-0016 . 27337224 PMC5066125

[33] Lomberk GA, Urrutia R. The Triple-Code Model for Pancreatic Cancer: Cross Talk Among Genetics, Epigenetics, and Nuclear Structure. The Surgical clinics of North America. 2015;95(5):935–52. Epub 2015/07/23. 10.1016/j.suc.2015.05.011 . 26315515 PMC4556141

[34] Sampath SC, Marazzi I, Yap KL, Sampath SC, Krutchinsky AN, Mecklenbräuker I, et al Methylation of a Histone Mimic within the Histone Methyltransferase G9a Regulates Protein Complex Assembly. Molecular Cell. 2007;27(4):596–608. 10.1016/j.molcel.2007.06.026 17707231

[35] Abe K, Naruse C, Kato T, Nishiuchi T, Saitou M, Asano M. Loss of Heterochromatin Protein 1 Gamma Reduces the Number of Primordial Germ Cells via Impaired Cell Cycle Progression in Mice1. Biology of Reproduction. 2011;85(5):1013–24. 10.1095/biolreprod.111.091512 21778144

[36] Akaike Y, Kuwano Y, Nishida K, Kurokawa K, Kajita K, Kano S, et al Homeodomain-interacting protein kinase 2 regulates DNA damage response through interacting with heterochromatin protein 1γ. Oncogene. 2015;34(26):3463–73. 10.1038/onc.2014.278 25151962

[37] Alam H, Li N, Dhar SS, Wu SJ, Lv J, Chen K, et al HP1γ Promotes Lung Adenocarcinoma by Downregulating the Transcription-Repressive Regulators NCOR2 and ZBTB7A. Cancer Res. 2018;78(14):3834–48. Epub 2018/05/15. 10.1158/0008-5472.CAN-17-3571 . 29764865 PMC6443096

[38] Bartkova J, Moudry P, Hodny Z, Lukas J, Rajpert-De Meyts E, Bartek J. Heterochromatin marks HP1γ, HP1α and H3K9me3, and DNA damage response activation in human testis development and germ cell tumours. International Journal of Andrology. 2011;34(4pt2):e103–e13. 10.1111/j.1365-2605.2010.01096.x 20695923

[39] Black JC, Allen A, Van Rechem C, Forbes E, Longworth M, Tschöp K, et al Conserved Antagonism between JMJD2A/KDM4A and HP1γ during Cell Cycle Progression. Molecular Cell. 2010;40(5):736–48. 10.1016/j.molcel.2010.11.008 21145482

[40] Bot C, Pfeiffer A, Giordano F, Manjeera DE, Dantuma NP, Ström L. Independent mechanisms recruit the cohesin loader protein NIPBL to sites of DNA damage. J Cell Sci. 2017;130(6):1134–46. Epub 2017/02/06. 10.1242/jcs.197236 . 28167679 PMC5358341

[41] Caillier M, Thénot S, Tribollet V, Birot A-M, Samarut J, Mey A. Role of the epigenetic regulator HP1γ in the control of embryonic stem cell properties. PloS one. 2010;5(11):e15507–e. 10.1371/journal.pone.0015507 21085495 PMC2981578

[42] Canudas S, Houghtaling BR, Bhanot M, Sasa G, Savage SA, Bertuch AA, et al A role for heterochromatin protein 1γ at human telomeres. Genes Dev. 2011;25(17):1807–19. Epub 2011/08/24. 10.1101/gad.17325211 . 21865325 PMC3175717

[43] Chang C, Liu J, He W, Qu M, Huang X, Deng Y, et al A regulatory circuit HP1γ/miR-451a/c-Myc promotes prostate cancer progression. Oncogene. 2018;37(4):415–26. 10.1038/onc.2017.332 28967902

[44] Grzenda A, Leonard P, Seo S, Mathison AJ, Urrutia G, Calvo E, et al Functional impact of Aurora A-mediated phosphorylation of HP1γ at serine 83 during cell cycle progression. Epigenetics Chromatin. 2013;6(1):21-. 10.1186/1756-8935-6-21 . 23829974 PMC3707784

[45] Huang C, Su T, Xue Y, Cheng C, Lay FD, McKee RA, et al Cbx3 maintains lineage specificity during neural differentiation. Genes Dev. 2017;31(3):241–6. 10.1101/gad.292169.116 . 28270516 PMC5358721

[46] Imanishi S, Umezu T, Kobayashi C, Ohta T, Ohyashiki K, Ohyashiki JH. Chromatin Regulation by HP1γ Contributes to Survival of 5-Azacytidine-Resistant Cells. Front Pharmacol. 2018;9:1166-. 10.3389/fphar.2018.01166 . 30386240 PMC6198088

[47] Kim H, Choi JD, Kim B-G, Kang HC, Lee J-S. Interactome Analysis Reveals that Heterochromatin Protein 1γ (HP1γ) Is Associated with the DNA Damage Response Pathway. Cancer Res Treat. 2016;48(1):322–33. Epub 2015/03/06. 10.4143/crt.2014.294 25761473 PMC4720079

[48] Kuwano Y, Nishida K, Akaike Y, Kurokawa K, Nishikawa T, Masuda K, et al Homeodomain-Interacting Protein Kinase-2: A Critical Regulator of the DNA Damage Response and the Epigenome. Int J Mol Sci. 2016;17(10):1638 10.3390/ijms17101638 . 27689990 PMC5085671

[49] Leonard PH, Grzenda A, Mathison A, Morbeck DE, Fredrickson JR, de Assuncao TM, et al The Aurora A-HP1γ pathway regulates gene expression and mitosis in cells from the sperm lineage. BMC Dev Biol. 2015;15:23-. 10.1186/s12861-015-0073-x . 26021315 PMC4448908

[50] Liu M, Huang F, Zhang D, Ju J, Wu X-B, Wang Y, et al Heterochromatin Protein HP1γ Promotes Colorectal Cancer Progression and Is Regulated by miR-30a. Cancer Res. 2015;75(21):4593 10.1158/0008-5472.CAN-14-3735 26333808

[51] Morikawa K, Ikeda N, Hisatome I, Shirayoshi Y. Heterochromatin protein 1γ overexpression in P19 embryonal carcinoma cells elicits spontaneous differentiation into the three germ layers. Biochem Biophys Res Commun. 2013;431(2):225–31. 10.1016/j.bbrc.2012.12.128 23313480

[52] Oka Y, Suzuki K, Yamauchi M, Mitsutake N, Yamashita S. Recruitment of the cohesin loading factor NIPBL to DNA double-strand breaks depends on MDC1, RNF168 and HP1γ in human cells. Biochem Biophys Res Commun. 2011;411(4):762–7. 10.1016/j.bbrc.2011.07.021 21784059

[53] Park J-W, Kim JJ, Bae Y-S. CK2 downregulation induces senescence-associated heterochromatic foci formation through activating SUV39h1 and inactivating G9a. Biochem Biophys Res Commun. 2018;505(1):67–73. 10.1016/j.bbrc.2018.09.099 30241941

[54] Sun M, Ha N, Pham D-H, Frederick M, Sharma B, Naruse C, et al Cbx3/HP1γ deficiency confers enhanced tumor-killing capacity on CD8(+) T cells. Sci Rep. 2017;7:42888-. 10.1038/srep42888 . 28220815 PMC5318867

[55] Takanashi M, Oikawa K, Fujita K, Kudo M, Kinoshita M, Kuroda M. Heterochromatin protein 1gamma epigenetically regulates cell differentiation and exhibits potential as a therapeutic target for various types of cancers. Am J Pathol. 2009;174(1):309–16. Epub 2008/12/04. 10.2353/ajpath.2009.080148 19056850 PMC2631343

[56] Wu W, Togashi Y, Johmura Y, Miyoshi Y, Nobuoka S, Nakanishi M, et al HP1 regulates the localization of FANCJ at sites of DNA double-strand breaks. Cancer Sci. 2016;107(10):1406–15. Epub 2016/09/01. 10.1111/cas.13008 . 27399284 PMC5084677

[57] Zhang H, Fu X, Su X, Yang A. CBX3/HP1γ is upregulated in tongue squamous cell carcinoma and is associated with an unfavorable prognosis. Exp Ther Med. 2018;15(5):4271–6. Epub 2018/03/20. 10.3892/etm.2018.5969 . 29731822 PMC5920882

[58] Zhang R, Chen W, Adams PD. Molecular dissection of formation of senescence-associated heterochromatin foci. Molecular and cellular biology. 2007;27(6):2343–58. Epub 2007/01/22. 10.1128/MCB.02019-06 17242207 PMC1820509

[59] Zhong X, Kan A, Zhang W, Zhou J, Zhang H, Chen J, et al CBX3/HP1γ promotes tumor proliferation and predicts poor survival in hepatocellular carcinoma. Aging (Albany NY). 2019;11(15):5483–97. Epub 2019/08/02. 10.18632/aging.102132 31375643 PMC6710055

[60] Zhou J, Bi H, Zhan P, Chang C, Xu C, Huang X, et al Overexpression of HP1γ is associated with poor prognosis in non-small cell lung cancer cell through promoting cell survival. Tumor Biology. 2014;35(10):9777–85. 10.1007/s13277-014-2182-8 24981246

[61] Bruns CJ, Harbison MT, Kuniyasu H, Eue I, Fidler IJ. In vivo selection and characterization of metastatic variants from human pancreatic adenocarcinoma by using orthotopic implantation in nude mice. Neoplasia. 1999;1(1):50–62. 10.1038/sj.neo.7900005 . 10935470 PMC1764837

