# Supplementary material for: Discovery, expression, cellular localization, and molecular properties of a novel, alternative spliced HP1γ isoform, lacking the chromoshadow domain
Source: PLoS One. 2020 Feb 6;15(2):e0217452. doi: 10.1371/journal.pone.0217452 (PMC7004349; doi:10.1371/journal.pone.0217452)
Supplement: S1 Table — (DOCX) [file pone.0217452.s001.docx]

| Bond Type | From Residues | To Residues |
| --- | --- | --- |
| Hydrogen-Donor; Positive | B:ALA1:HT1 | :GLU33:OE2 |
| Positive | B:M3L9:NZ | :GLU26:OE2 |
| Hydrogen-Donor | :LYS11A:HZ1 | B:GLY13:O |
| Hydrogen-Donor | :LYS11A:HZ3 | B:GLY12:O |
| Hydrogen-Donor | :LYS21:HZ2 | B:SER10:OG |
| Hydrogen-Donor | :LYS21:HZ2 | B:SER10:O |
| Hydrogen-Donor | :ALA25:HN | B:SER10:OG |
| Hydrogen-Donor | :PHE30:HN | B:ALA7:O |
| Hydrogen-Donor | :LYS52:HZ1 | B:ARG2:O |
| Hydrogen-Donor | :LYS52:HZ2 | B:THR3:O |
| Hydrogen-Donor | :LYS52:HZ3 | B:ALA1:O |
| Hydrogen-Donor | :LYS52:HZ3 | B:THR3:OG1 |
| Hydrogen-Donor | :ASN68:HD22 | B:ARG8:O |
| Hydrogen-Donor | :ASN68:HD22 | B:M3L9:O |
| Hydrogen-Donor | B:GLN5:HE21 | :GLU33:O |
| Hydrogen-Donor | B:GLN5:HE22 | :ASN74:OD1 |
| Hydrogen-Donor | B:ALA7:HN | :PHE30:O |
| Hydrogen-Donor | B:ARG8:HN | :ASN68:O |
| Hydrogen-Donor | B:ARG8:HH11 | :TYR70:OH |
| Hydrogen-Donor | B:ARG8:HH21 | :ASN68:OD1 |
| Hydrogen-Donor | B:ARG8:HH22 | :GLU67:O |
| Hydrogen-Donor | B:M3L9:HN | :GLU28:O |
| Hydrogen-Donor | B:SER10:HN | :ALA25:O |
| Hydrogen-Donor | B:SER10:HG | :GLU23:OE1 |
| Hydrogen-Donor | B:SER10:HG | :GLU24:O |
| Hydrogen-Donor | :GLY12A:HA1 | B:GLY12:O |
| Hydrogen-Donor | :LYS21:HE2 | B:SER10:O |
| Hydrogen-Donor | :GLU29:HA | B:ALA7:O |
| Hydrogen-Donor | B:THR3:HB | :GLU33:O |
| Hydrogen-Donor | B:THR6:HA | :PHE30:O |
| Hydrogen-Donor | B:THR6:HB | :GLU29:OE2 |
| Hydrogen-Donor | B:ARG8:HA | :GLU28:O |
| Hydrogen-Donor | B:ARG8:HD1 | :TYR70:OH |
| Hydrogen-Donor | B:M3L9:HM23 | :GLU26:OE2 |
| Hydrogen-Donor | B:M3L9:HM31 | :LYS60:O |
| Hydrogen-Donor | B:GLY13:HA1 | :MET12:O |
| Hydrogen-Donor | B:ALA15:HA | :ASN68:OD1 |
| Positive | B:M3L9:NZ | :PHE30 |
| Positive | B:M3L9:NZ | :TRP51 |
| Positive | B:M3L9:NZ | :TRP51 |
| Alkyl | :VAL31 | B:LYS4 |
| Alkyl | B:ALA7 | :VAL32 |
| Alkyl | B:ALA7 | :LEU69 |
| Alkyl | B:ALA7 | :LEU71 |
| Pi-Orbitals | :TRP51 | B:ALA7 |

**S1 Table: Additional bonds that further stabilize sHP1γ – Histone 3 tail complex.**
