# Supplementary material for: Discovery, expression, cellular localization, and molecular properties of a novel, alternative spliced HP1γ isoform, lacking the chromoshadow domain
Source: PLoS One. 2020 Feb 6;15(2):e0217452. doi: 10.1371/journal.pone.0217452 (PMC7004349; doi:10.1371/journal.pone.0217452)
Supplement: S1 Fig — Using the GTEx dataset, we show the distribution of gene expression for the sHP1γ isoform, for each body tissue and using smoothed density (violin) plots. The protein is expressed at a low level in most human tissues. Tissues are colored tan for brain regions, dark red for primary gastrointestinal track, red for arterial and cardiac tissues, and pink for all others. (DOCX) [file pone.0217452.s002.docx]

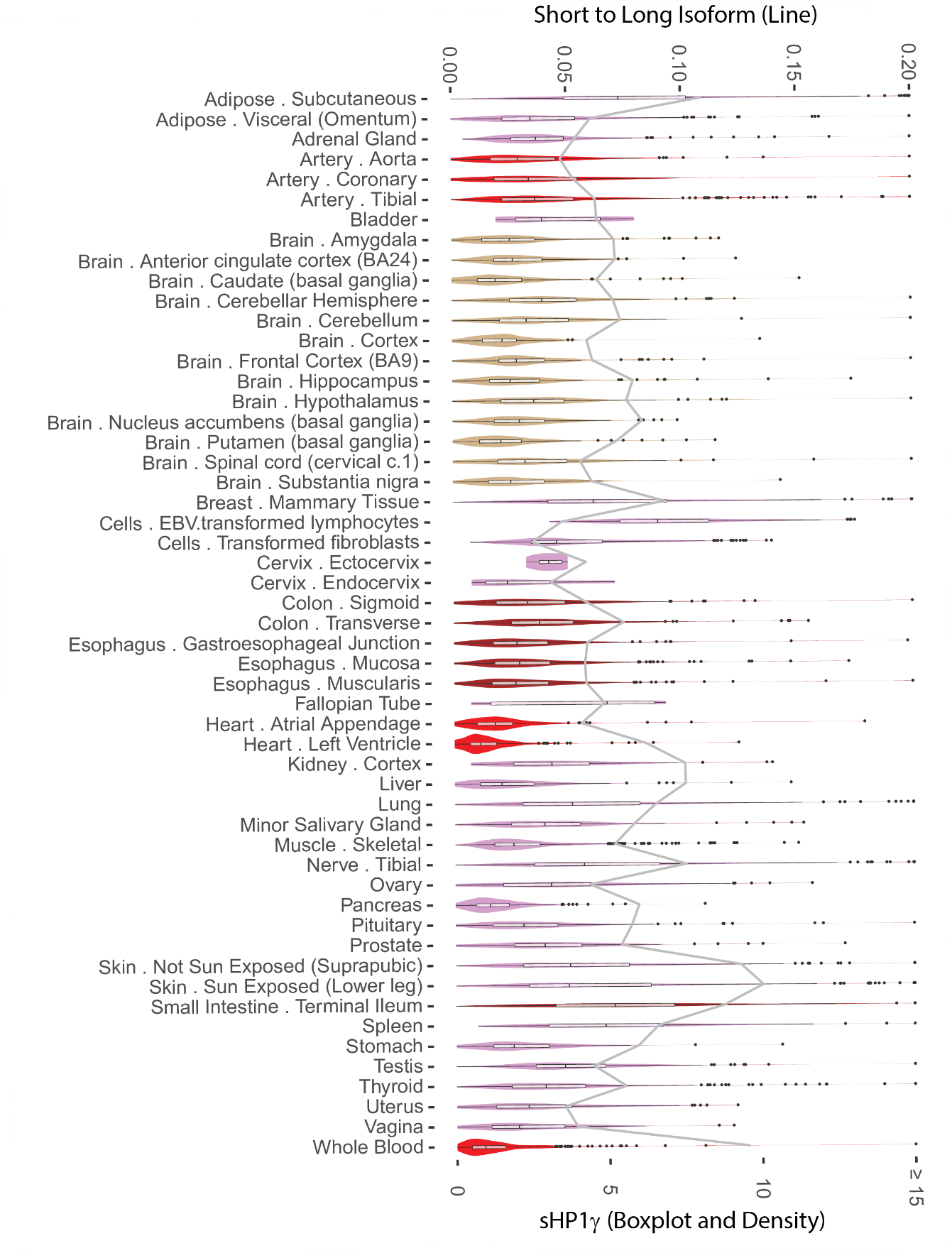


**S1 Fig.** **sHP1γ has variable expression across normal human tissues.**

Using the GTEx dataset, we show the distribution of gene expression for the sHP1γ isoform, for each body tissue and using smoothed density (violin) plots. The protein is expressed at a low level in most human tissues. Tissues are colored tan for brain regions, dark red for primary gastrointestinal track, red for arterial and cardiac tissues, and pink for all others.
