# Supplementary material for: Discovery, expression, cellular localization, and molecular properties of a novel, alternative spliced HP1γ isoform, lacking the chromoshadow domain
Source: PLoS One. 2020 Feb 6;15(2):e0217452. doi: 10.1371/journal.pone.0217452 (PMC7004349; doi:10.1371/journal.pone.0217452)
Supplement: S2 Fig — Using TCGA data, we show the ratio of short to long isoform for each cancer type, designating each cancer type by its official abbreviation. A horizontal line marks the median value across all samples. Cancer types are colored by their median value. Many cancer types have the majority of their samples below the global median, while others have many samples above the global median, demonstrating that the two isoforms are likely regulated in different ways by cancers of different tissues. (DOCX) [file pone.0217452.s003.docx]

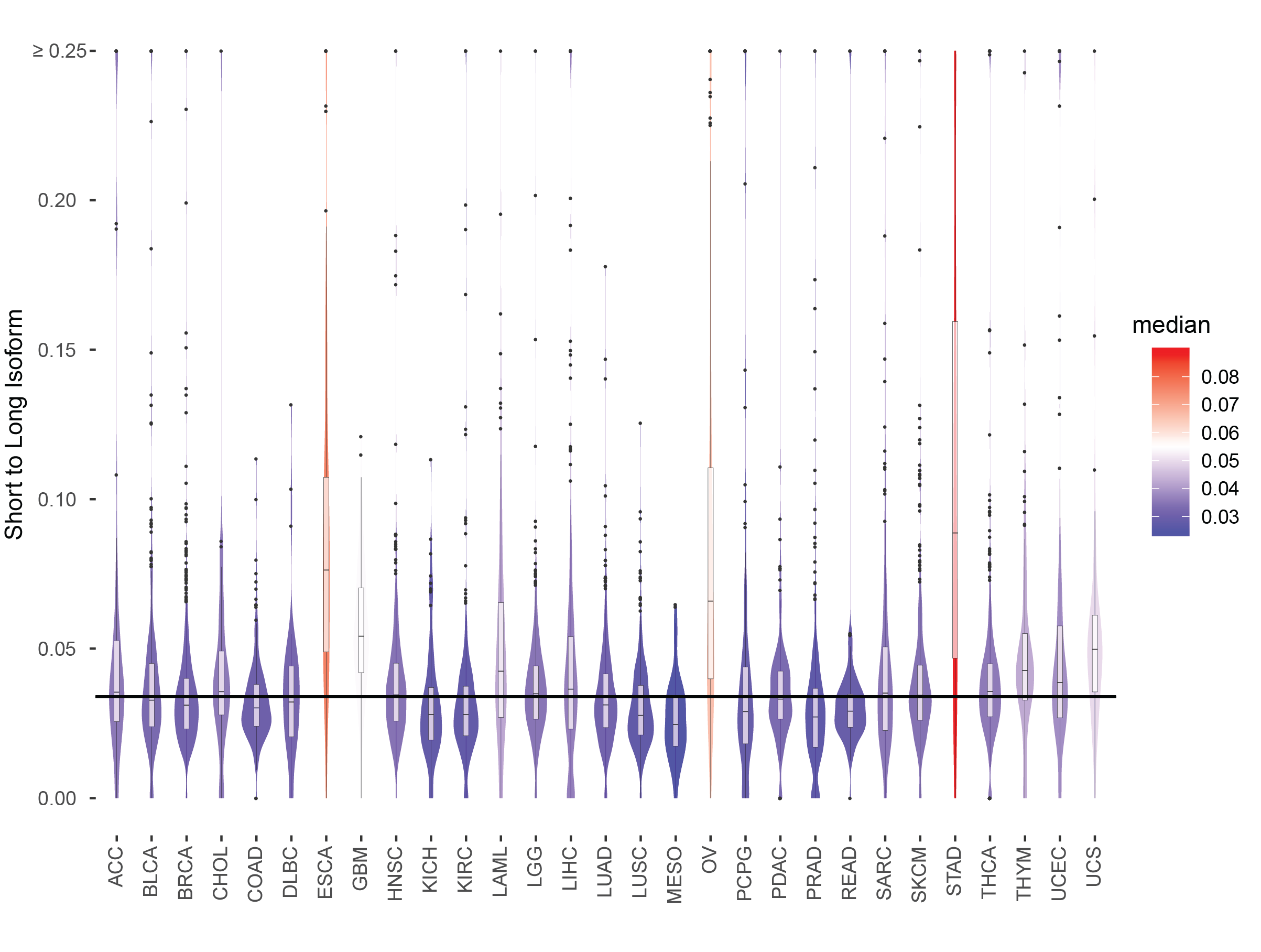


**S2 Fig. sHP1γ is highly expressed in a subset of nearly all tumors.**

Using TCGA data, we show the ratio of short to long isoform for each cancer type, designating each cancer type by its official abbreviation. A horizontal line marks the median value across all samples. Cancer types are colored by their median value. Many cancer types have the majority of their samples below the global median, while others have many samples above the global median, demonstrating that the two isoforms are likely regulated in different ways by cancers of different tissues.
