# Supplementary material for: Discovery, expression, cellular localization, and molecular properties of a novel, alternative spliced HP1γ isoform, lacking the chromoshadow domain
Source: PLoS One. 2020 Feb 6;15(2):e0217452. doi: 10.1371/journal.pone.0217452 (PMC7004349; doi:10.1371/journal.pone.0217452)
Supplement: S3 Fig — (A) Lysates from CHO cells transfected with empty vector, His-sHP1γ and His-HP1γ were used for Western blot analyses with a newly generated peptide-specific antibody against sHP1γ. The overexpression of both HP1γ isoforms was confirmed with His antibody and β-actin was used as reference control. Molecular weight markers on the left illustrate the size difference of the two HP1γ isoforms. The cropped images for lanes 5–7 shown in Fig 4A. Lanes 1–4 and 8–9 were samples for an irrelevant study. (B) Lysates from five pancreatic cell lines were used for Western blot to analysis the relative expression of sHP1γ and HP1γ. Total H3 antibody was used as a loading control. The cropped images are presented in Fig 4B. (C) Western blot of purified sHP1γ and HP1γ proteins probed with an antibody to the N-terminus of HP1γ to recognize both proteins, simultaneously. The cropped images are shown in Fig 7C. (DOCX) [file pone.0217452.s004.docx]

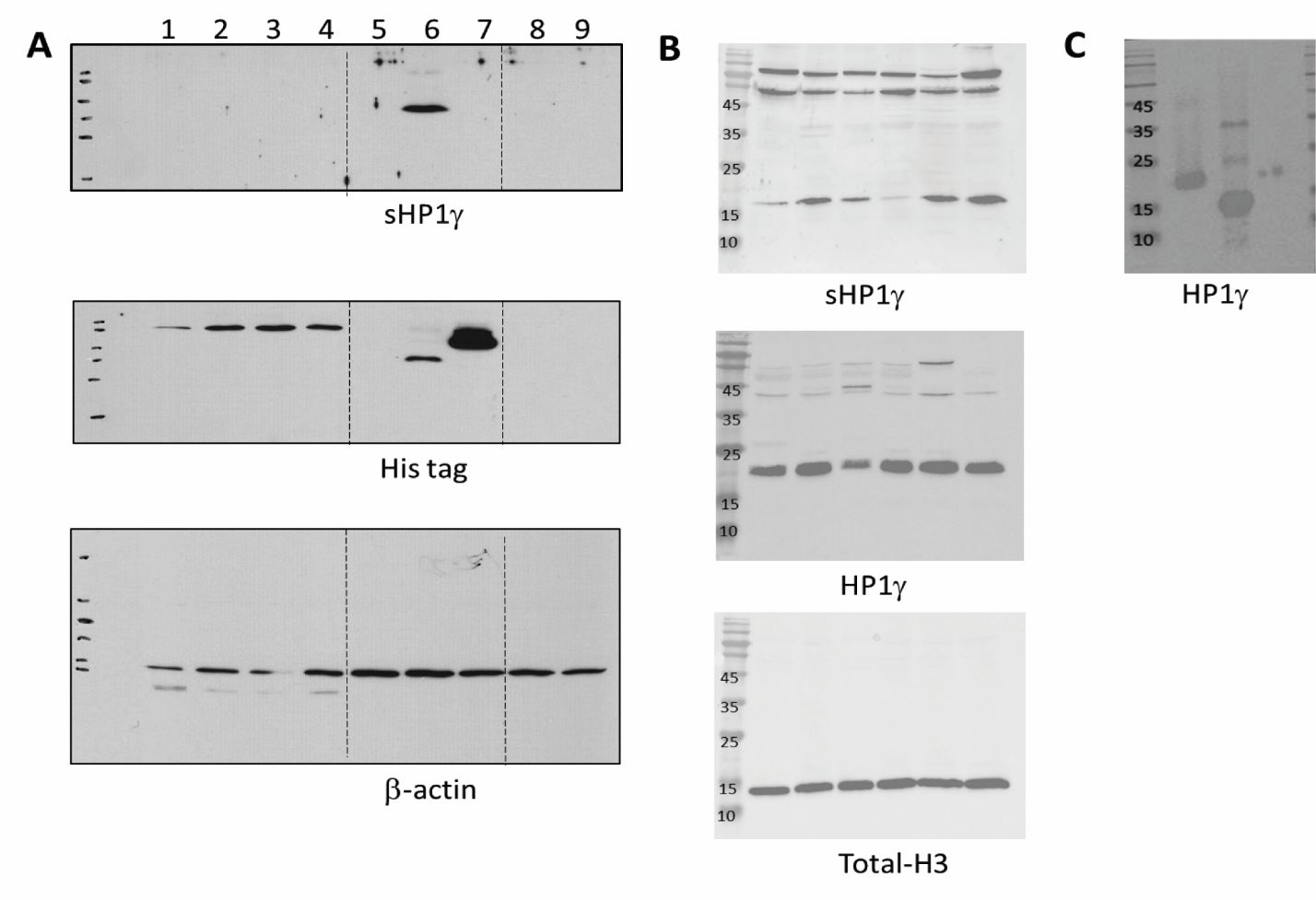


**S3 Fig. Full length Western Blot images from Figures 4A, 4B and 7C.**

(A) Lysates from CHO cells transfected with empty vector, His-sHP1γ and His-HP1γ were used for Western blot analyses with a newly generated peptide-specific antibody against sHP1γ. The overexpression of both HP1γ isoforms was confirmed with His antibody and β-actin was used as reference control. Molecular weight markers on the left illustrate the size difference of the two HP1γ isoforms. The cropped images for lanes 5-7 shown in Figure 4A. Lanes 1-4 and 8-9 were samples for an irrelevant study. (B) Lysates from five pancreatic cell lines were used for Western blot to analysis the relative expression of sHP1γ and HP1γ. Total H3 antibody was used as a loading control. The cropped images are presented in Figure 4B. (C) Western blot of purified sHP1γ and HP1γ proteins probed with an antibody to the N-terminus of HP1γ to recognize both proteins, simultaneously. The cropped images are shown in Figure 7C.
