# Supplementary material for: Discovery, expression, cellular localization, and molecular properties of a novel, alternative spliced HP1γ isoform, lacking the chromoshadow domain
Source: PLoS One. 2020 Feb 6;15(2):e0217452. doi: 10.1371/journal.pone.0217452 (PMC7004349; doi:10.1371/journal.pone.0217452)
Supplement: S4 Fig — (A) 3D structure of sHP1γ as modelled by I-TASSER, (B) 3D structure of sHP1γ generated using X-Raptor. Note that both are remarkably similar to each other and to the homology-based model depicted in Fig 6A. (DOCX) [file pone.0217452.s005.docx]

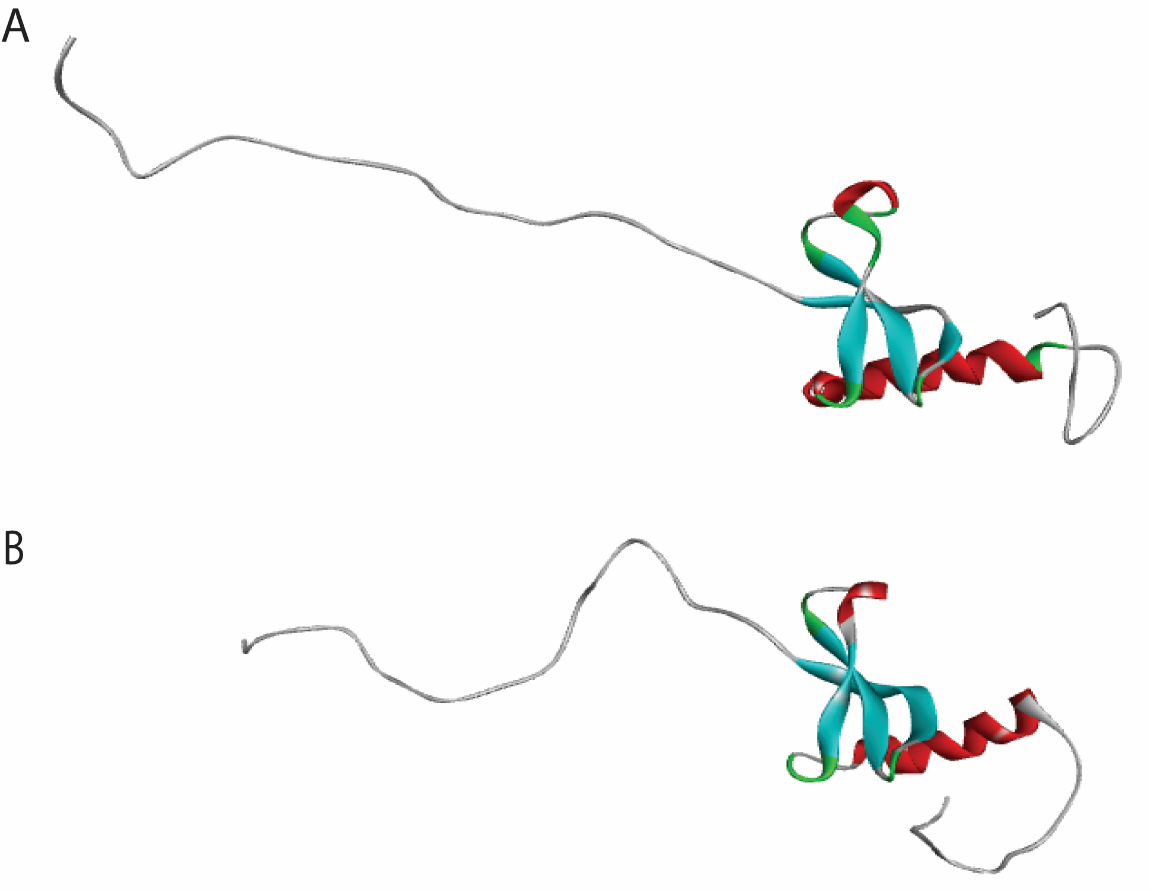


**S4 Fig.** **Sequence-to-Structure Prediction by High CASP Performer Algorithms.**

(A) 3D structure of sHP1γ as modelled by I-TASSER, (B) 3D structure of sHP1γ generated using X-Raptor. Note that both are remarkably similar to each other and to the homology-based model depicted in Fig.6A.
