# Supplementary material for: Discovery, expression, cellular localization, and molecular properties of a novel, alternative spliced HP1γ isoform, lacking the chromoshadow domain
Source: PLoS One. 2020 Feb 6;15(2):e0217452. doi: 10.1371/journal.pone.0217452 (PMC7004349; doi:10.1371/journal.pone.0217452)
Supplement: S5 Fig — The quality of the structural model is high in that 96% of its residues are present in the expected region. (DOCX) [file pone.0217452.s006.docx]

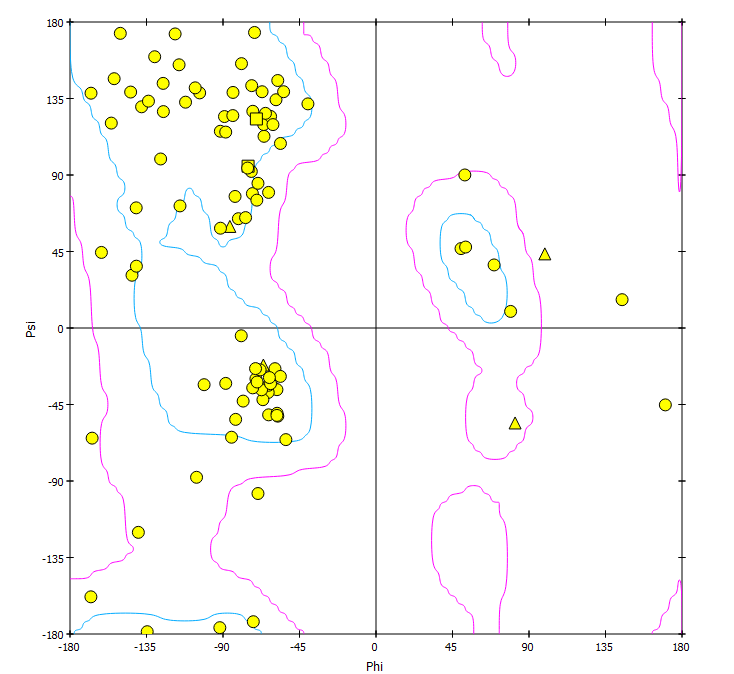


**S5 Fig.** **Ramachandran plot of sHP1γ.**

The quality of the structural model is high in that 96% of its residues are present in the expected region.
