# Supplementary material for: Discovery, expression, cellular localization, and molecular properties of a novel, alternative spliced HP1γ isoform, lacking the chromoshadow domain
Source: PLoS One. 2020 Feb 6;15(2):e0217452. doi: 10.1371/journal.pone.0217452 (PMC7004349; doi:10.1371/journal.pone.0217452)
Supplement: S6 Fig — Molecular dynamics (MD) simulations (5 ns), shown in duplicate, comparing the peptide bound form (Holo) to the non-peptide bound form (Apo) of sHP1γ. Note that binding to the H3K9Me3 histone mark-containing peptide stabilizes the complex and reduces the intrinsic flexibility of the chromodomain. (DOCX) [file pone.0217452.s007.docx]

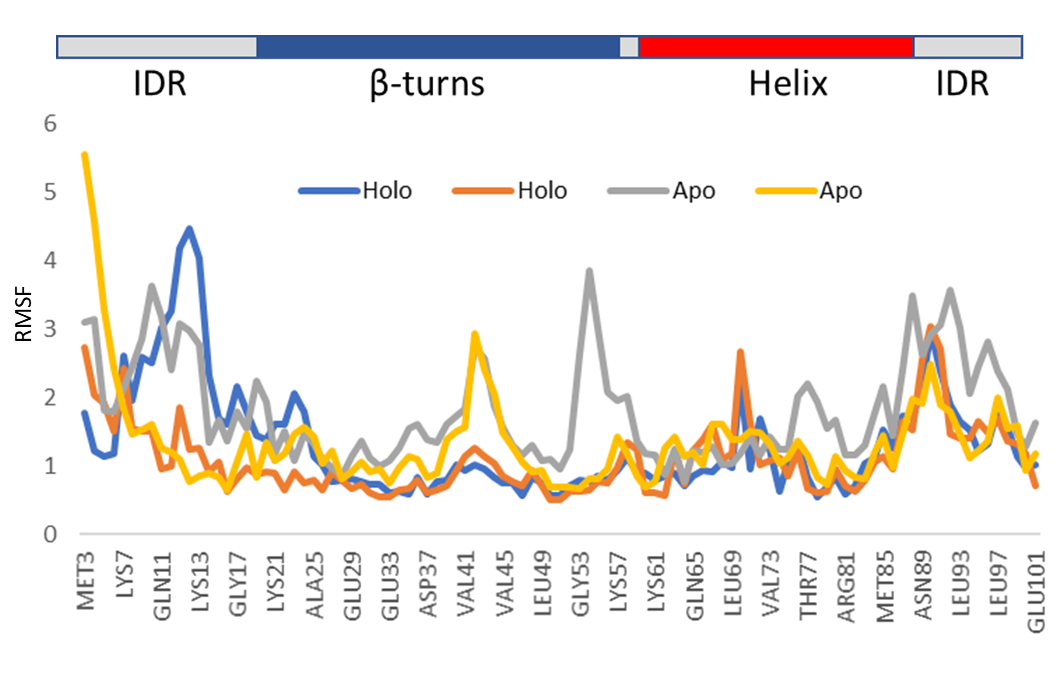


**S6 Fig.** **RMSF Values of H3K9Me3-Bound and Unbound sHP1γ.**

Molecular dynamics (MD) simulations (5 ns), shown in duplicate, comparing the peptide bound form (Holo) to the non-peptide bound form (Apo) of sHP1γ. Note that binding to the H3K9Me3 histone mark-containing peptide stabilizes the complex and reduces the intrinsic flexibility of the chromodomain.
